# Long-term impact of maternal high-fat diet on offspring cardiac health: role of micro-RNA biogenesis

**DOI:** 10.1038/s41420-019-0153-y

**Published:** 2019-03-01

**Authors:** Benazir Siddeek, Claire Mauduit, Hassib Chehade, Guillaume Blin, Marjorie Liand, Mariapia Chindamo, Mohamed Benahmed, Umberto Simeoni

**Affiliations:** 1 Woman-Mother-Child Department, Division of Pediatrics, DOHaD Laboratory, Centre Hospitalier Universitaire Vaudois and University of Lausanne, Lausanne, Switzerland; 2 INSERM U1065, Centre Méditerranéen de Médecine Moléculaire (C3M), Team 5, Nice, France

## Abstract

Heart failure is a worldwide leading cause of death. Diet and obesity are particularly of high concern in heart disease etiology. Gravely, altered nutrition during developmental windows of vulnerability can have long-term impact on heart health; however, the underlying mechanisms are poorly understood. In the understanding of the initiation of chronic diseases related to developmental exposure to environmental challenges, deregulations in epigenetic mechanisms including micro-RNAs have been proposed as key events. In this context, we aimed at delineating the role of micro-RNAs in the programming of cardiac alterations induced by early developmental exposure to nutritional imbalance. To reach our aim, we developed a human relevant model of developmental exposure to nutritional imbalance by maternally exposing rat to high-fat diet during gestation and lactation. In this model, offspring exposed to maternal high-fat diet developed cardiac hypertrophy and increased extracellular matrix depot compared to those exposed to chow diet. Microarray approach performed on cardiac tissue allowed the identification of a micro-RNA subset which was down-regulated in high-fat diet-exposed animals and which were predicted to regulate transforming growth factor-beta (TGFβ)-mediated remodeling. As indicated by in vitro approaches and gene expression measurement in the heart of our animals, decrease in DiGeorge critical region 8 (DGCR8) expression, involved in micro-RNA biogenesis, seems to be a critical point in the alterations of the micro-RNA profile and the TGFβ-mediated remodeling induced by maternal exposure to high-fat diet. Finally, increasing DGCR8 activity and/or expression through hemin treatment in vitro revealed its potential in the rescue of the pro-fibrotic phenotype in cardiomyocytes driven by DGCR8 decrease. These findings suggest that cardiac alterations induced by maternal exposure to high-fat diet is related to abnormalities in TGFβ pathway and associated with down-regulated micro-RNA processing. Our study highlighted DGCR8 as a potential therapeutic target for heart diseases related to early exposure to dietary challenge.

## Introduction

A worldwide upward trend in the burden of non-communicable diseases such as cancers, stroke, heart and chronic lung diseases is currently observed. These diseases share common risks factors that are related to lifestyle including smoking, physical inactivity, alcohol and unhealthy diet consumption (World Health Organization (WHO); https://www.who.int/ncds/en/). Importantly, these factors have even more serious impact on health when the exposure occurs during developmental windows of vulnerability such as in utero and early post-natal life. As such, maternal overweight and obesity, the incidence of which are increasing (WHO; http://www.who.int/en/news-room/fact-sheets/detail/obesity-and-overweight) and which are often attributed to excessive intake of calorie-dense food, in particular high-fat diets, and reduced physical activity^1^, not only have consequences in the short term with pregnancy complications, but also impact offspring health later during life. Maternal obesity and dietary imbalances are particularly of high concern in the early origins of heart failure^2,3^. Since human cardiomyocyte have limited proliferative capacity, the exposure of the developing heart to environmental challenges such as nutritional excess may have deleterious effects on lifelong cardiomyocyte functions^4^. Indeed, maternal exposure to high-fat diet and maternal obesity increases the risk for impaired cardiac function in adulthood by modifying heart rate and inducing left ventricular wall thickness, hypertrophy^5^ and fibrosis^6,7^. In the early origins of heart diseases, epigenetic mechanisms are likely key players. In particular, micro-RNAs, which belong to small non-coding RNAs (around 22 nucleotides in length) involved in each step of heart development, seem to play a crucial role in the unfavorable programming of heart diseases^8^. By controlling about 60% of all protein-coding genes in mammals, micro-RNAs are considered as significant post-transcriptional regulators of gene expression^9^, and regulate almost all biological processes including development, differentiation, proliferation, apoptosis, metabolism and tissue remodeling. The critical role of maintaining micro-RNA balance in the heart has been revealed notably in models of mice where cardiomyopathies were induced by inhibiting the expression of proteins involved in micro-RNA biogenesis in cardiomyocytes^10–12^. Micro-RNA biogenesis is a regulated multistep process starting in the cell nucleus where primary micro-RNAs (pri-micro-RNAs) are transcribed mainly by RNA polymerase II. Through the action of a microprocessor complex including nuclear ribonuclease III (DROSHA) and its cofactor DiGeorge critical region 8 (DGCR8), pri-micro-RNAs are trimmed into 70 nucleotide hairpins called precursor micro-RNAs (pre-micro-RNAs)^13^. Then, pre-micro-RNAs assembles into a complex constituted by Exportin-5 (XPO5) and RanGTP and translocate into the cytoplasm^14^ where they continue their maturation, through a splicing realized by a complex including DICER and TAR RNA-binding protein 2 (TRBP). This results in RNA duplexes comprising the mature micro-RNAs and the passenger strand, micro-RNAs*^15^. Finally, each mature single strand micro-RNA strand is incorporated into the RNA-induced silencing complex (RISC) comprising an Argonaute (AGO) protein to direct the silencing of the targeted mRNA^16^. Interestingly, the micro-RNA biogenesis machinery is sensitive to hormonal regulation and dietary changes^17–19^. In this context, micro-RNAs and their regulation represent a potential candidate in the understanding of the long-term effects on cardiac functions induced by exposure to nutritional challenges early during development. Thus, based on a human relevant model of cardiac alterations programmed by exposure to maternal high-fat diet, we aimed at delineating the role of micro-RNAs, identifying the defects in the micro-RNA biogenesis machinery in such programming and at highlighting potential targets and approaches for the phenotype reversal.

## Results

### Exposure to maternal high-fat diet induces cardiac hypertrophy and increases cardiac extracellular matrix depot

**Fig. 1:**
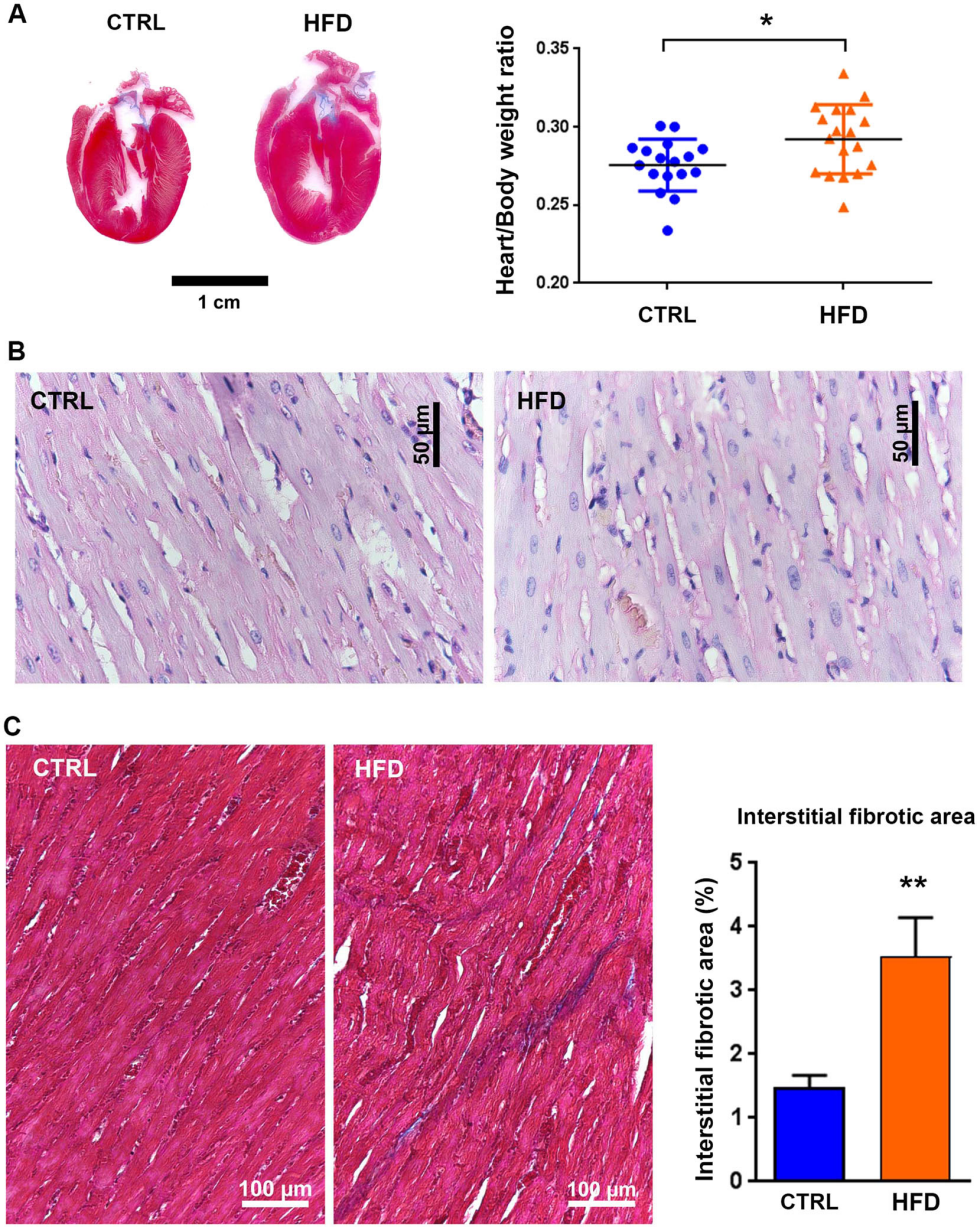
Effects of maternal exposure to high-fat diet on adult heart. (**a**) Heart vs. body weight ratio of animals exposed to chow diet (CTRL) or high-fat diet (HFD). Data are expressed as mean ± SEM; *n* = 18 per group. Representative heart section image from a male rat at post-natal day 77 under control chow diet (CTRL) or HFD stained with hematoxylin–eosin staining for cell morphology analyses (scale bar = 50 μm) (**b**) and Masson’s trichrome to evaluate extracellular matrix depot (scale bar = 100 μm) (**c**). *P < 0.05 and **P < 0.01 compared to control

At post-natal day 77 (PND77), whereas no difference was detected in the body weight of animals exposed to chow diet (CTRL) or to high-fat diet (HFD) (supplementary data S1), increased heart vs. body weight ratio was induced by maternal exposure to high-fat diet (Fig. 1a). This was associated with enlarged cardiomyocytes visualized by hematoxylin–eosin staining (Fig. 1b). In addition, exposure to high-fat diet increased extracellular matrix depot as indicated by Masson’s trichrome staining (Fig. 1c). These data indicate that maternal exposure to high-fat diet induced cardiac remodeling with hypertrophic and fibrotic patterns, without changes in the body weight at PND77.

### Exposure to maternal high-fat diet alters adult cardiac micro-RNA levels

**Fig. 2:**
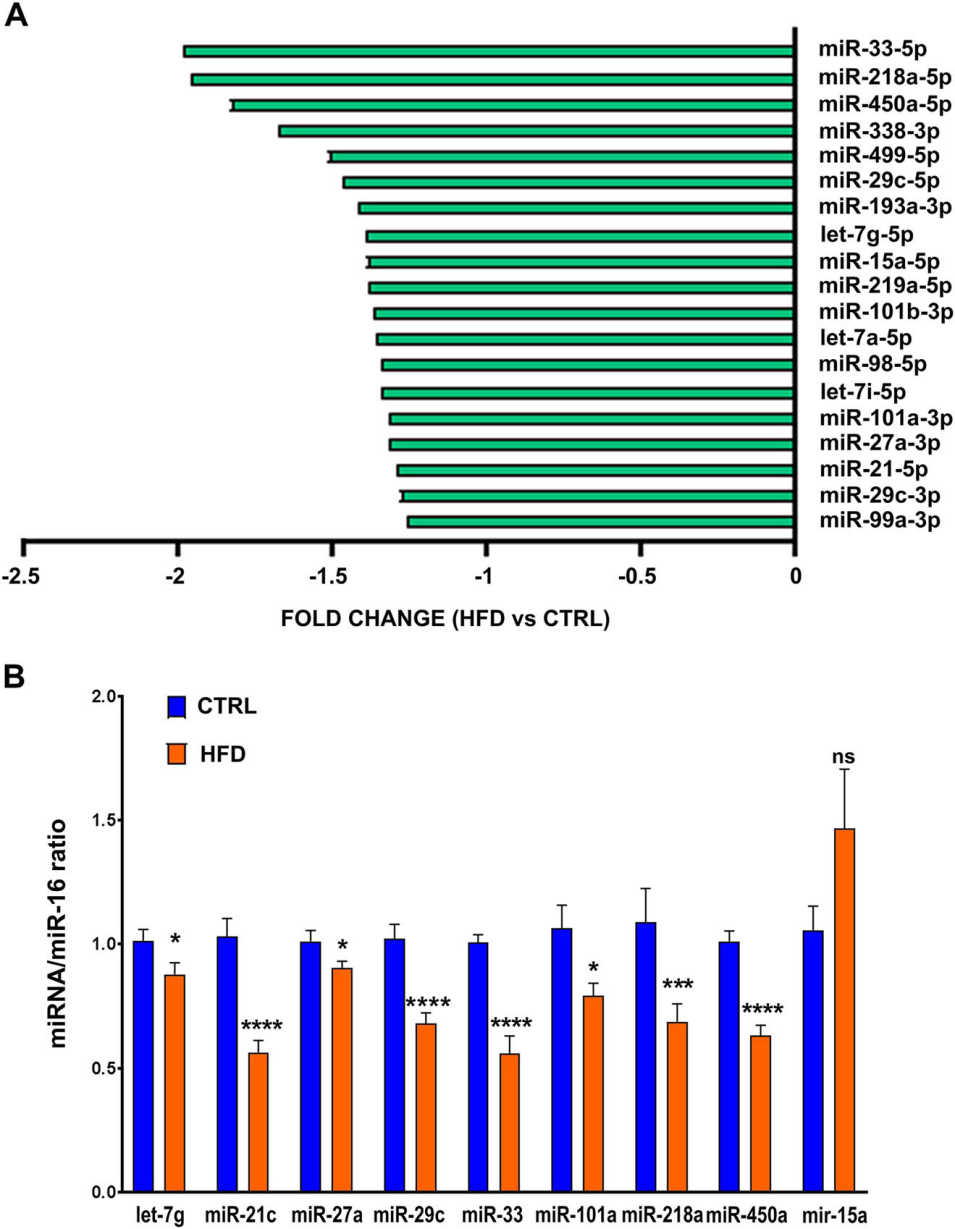
Effects of maternal exposure to high-fat diet on adult cardiac micro-RNA levels. (**a**) Differential expression of micro-RNAs detected by microarray in the heart of male rats at post-natal day 77 exposed to maternal high-fat diet compared to chow diet. (**b**) let-7g, miR-15, miR-21, miR-27a, miR-29c, miR-33, miR-101a, miR-218a and miR-450a levels were analyzed by quantitative real-time PCR (RT-qPCR) in the heart of male rats at post-natal day 77 under control chow diet (CTRL) or high-fat diet (HFD). Data are expressed as mean ± SEM; *n* = 13 per group. *P < 0.05, ***P < 0.001 and ****P < 0.0001 compared to control, ns not statistically significant

**Fig. 3:**
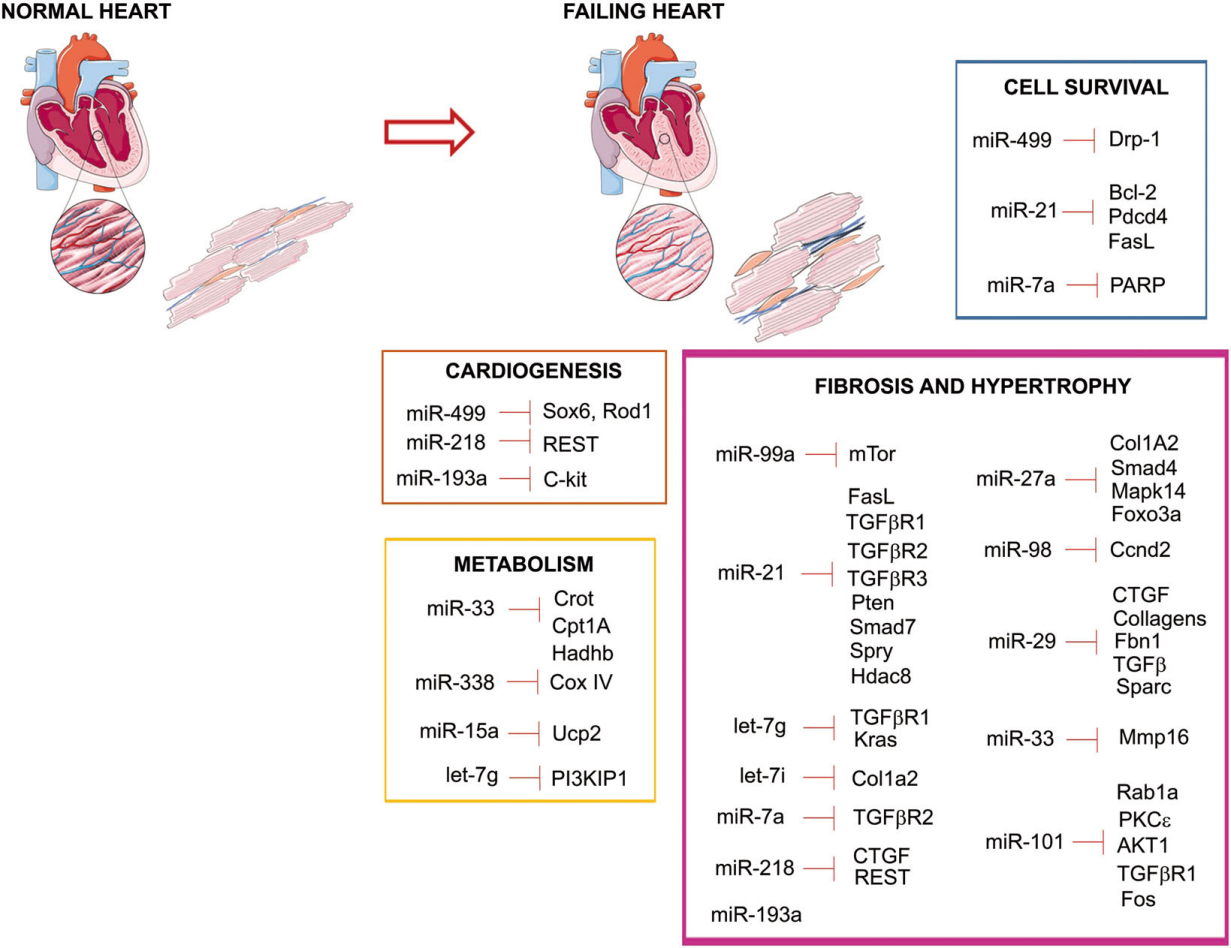
Role of the down-regulated micro-RNAs in heart disease. Scheme representing the putative targets of the micro-RNAs down-regulated by maternal exposure to high-fat diet, reported in heart diseases related to the regulation of metabolism, cardiogenesis, cell survival, hypertrophy and fibrosis

Since micro-RNAs have been described as effectors of environmental influences on gene expression and disease^20^, and because alterations in micro-RNA balance have been reported to induce cardiac hypertrophy and fibrosis, we wondered what could be the role of micro-RNAs in the cardiac tissular alterations induced by maternal exposure to high-fat diet. With such an aim, we analyzed the micro-RNA profile by microarray experiment. The microarray quality control data are presented in supplemental data S2. The comparison of micro-RNA profile between rats exposed to chow diet (CTRL) or to HFD highlighted 19 down-regulated micro-RNAs, with an adjusted *P* value < 0.05 (Fig. 2a). Nine micro-RNAs (let-7g, miR-15, miR-21, miR-27a, miR-29c, miR-33, miR-101a, miR-218a and miR-450a) were selected for quantitative real-time PCR (RT-qPCR) validation of the microarray data (Fig. 2b). Except for miR-15 where no modification was detected, RT-qPCR data correlated well with those obtained from the microarray for all selected micro-RNAs, confirming the down-regulation of micro-RNA levels in the heart of animals exposed to maternal high-fat diet compared to the ones exposed to chow diet. All the 19 significantly differentially expressed micro-RNAs have previously been reported in experimental or human cardiovascular diseases (supplemental table 2). The targets of the altered micro-RNAs have been reported to play a role in metabolism, cardiogenesis, cell survival, hypertrophy and fibrosis (Fig. 3). Collectively, these data suggest that tissular cardiac alterations observed in the heart of animals exposed to maternal high-fat diet are linked to the down-regulation of a micro-RNA subset regulating cellular mechanisms that are critical for cardiac functions.

### Altered cardiac micro-RNAs are involved in cardiac diseases and associated pathways

**Fig. 4:**
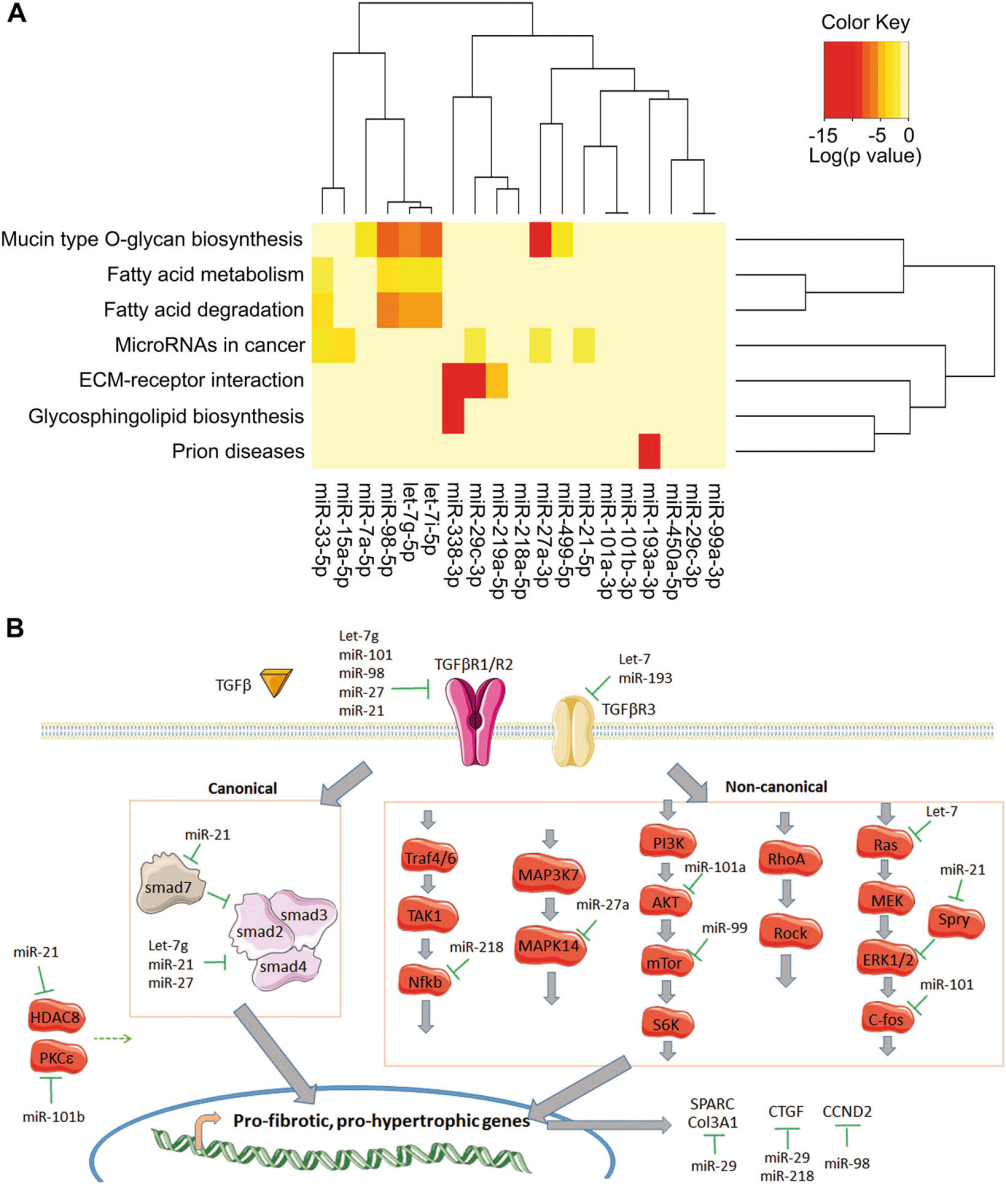
Effects of maternal exposure to high-fat diet on the expression of genes involved in transforming growth factor-beta (TGFβ)-regulated hypertrophy and fibrosis. (**a**) Cluster heat maps showing the pathways which are predicted to be altered based on significance levels. The more intense red color indicates higher probability that a specific pathway is significantly enriched with target genes for a certain micro-RNA. (**b**) Scheme representing the involvement of down-regulated micro-RNAs in the regulation of canonical and non-canonical TGFβ pathways. Putative inhibition of genes by micro-RNAs is represented by green line. Dotted lines represent direct or indirect stimulation of the pathway

To identify regulatory networks involved in the adult heart dysfunctions induced by maternal exposure to high-fat diet, we performed a computational target prediction analysis coupled to pathway analysis using DIANA-miRPath (v3.0) software. Notably, KEGG (Kyoto Encyclopedia of Genes and Genomes) pathway analysis showed that the altered micro-RNAs are predicted to target extracellular matrix–receptor interaction, fatty acid degradation and metabolism. The DIANA-miRPath software calculates the significance for all the miRNA–mRNA pairs in a pathway, and then combines them into a merged *P* value for each pathway^21^. The results are reported as heat maps, and the pathways are clustered based on significance levels (Fig. 4a). A closer look at the altered micro-RNA targets revealed that the majority of the altered micro-RNAs are involved in transforming growth factor-beta (TGFβ) signaling which is closely linked to extracellular matrix–receptor interaction and involved in cardiac fibrosis and hypertrophy (Fig. 4b). In the response to cardiac stress, TGFβ is subsequently activated and triggers a cascade of signaling involving SMAD protein phosphorylation (canonical pathway) or the activation of other intermediates such as mechanistic target of rapamycin (mTOR) or mitogen-activated protein kinase (MAPK) (non-canonical pathway), leading to the up-regulation of the expression of genes involved notably in the regulation of extracellular matrix synthesis and cell growth (Fig. 4b). As such, the dysregulation of TGFβ pathway has been shown to result in fibrosis and hypertrophy^22^. In light of these points, we supposed that the cardiac hypertrophy and fibrosis induced by maternal exposure to high-fat diet are due to micro-RNA-mediated TGFβ pathway dysregulation.

### Exposure to maternal high-fat diet alters the expression of genes involved in TGFβ regulated hypertrophy and fibrosis

**Fig. 5:**
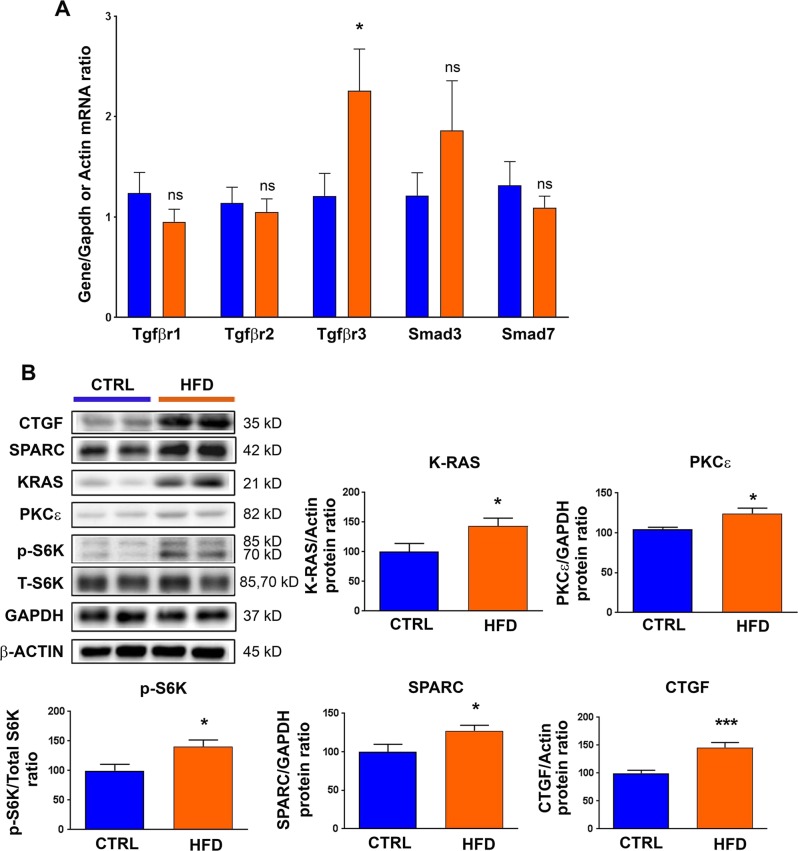
Effects of maternal exposure to high-fat diet on the expression of genes involved in transforming growth factor-beta (TGFβ)-regulated hypertrophy and fibrosis. (**a**) mRNA and (**b**) protein levels of putative target genes involved in TGFβ-mediated remodeling were analyzed in the heart from male rats at post-natal day 77 under control chow diet (CTRL) or high-fat diet (HFD). In (**b**), a representative immunoblot image is presented for each protein. Data are expressed as the mean ± SEM; *n* = 13 per group. *P < 0.05, and ***P < 0.001 compared to control, ns not statistically significant

To verify whether the micro-RNA down-regulation was associated with alterations in the TGFβ pathway in the heart of animals exposed to maternal high-fat diet, we measured the expression levels of the predicted targets of these micro-RNAs, involved in TGFβ-mediated fibrosis and hypertrophy. Effectively, in the heart of animals exposed to high-fat diet compared to chow diet, increased transforming growth factor-beta receptor type III (TGFβR3) mRNA levels were detected (Fig. 5a). This was associated with increased expression of pro-hypertrophic genes such as V-Ki-ras2 Kirsten rat sarcoma viral oncogene homolog (K-RAS), protein kinase C epsilon (PRKCE), increased phosphorylation of ribosomal protein s6 kinase (RPS6KB1, a target of mTOR), and increased expression of pro-fibrotic genes such as connective tissue growth factor (CTGF) and secreted protein acidic cysteine-rich (SPARC) (Fig. 5b). These data suggest that cardiac hypertrophy and fibrosis induced by maternal exposure to high-fat diet might be due to micro-RNA-mediated TGFβ pathway dysregulation at multiple levels.

### Maternal exposure to high-fat diet modifies the expression of proteins involved in micro-RNA processing

**Fig. 6:**
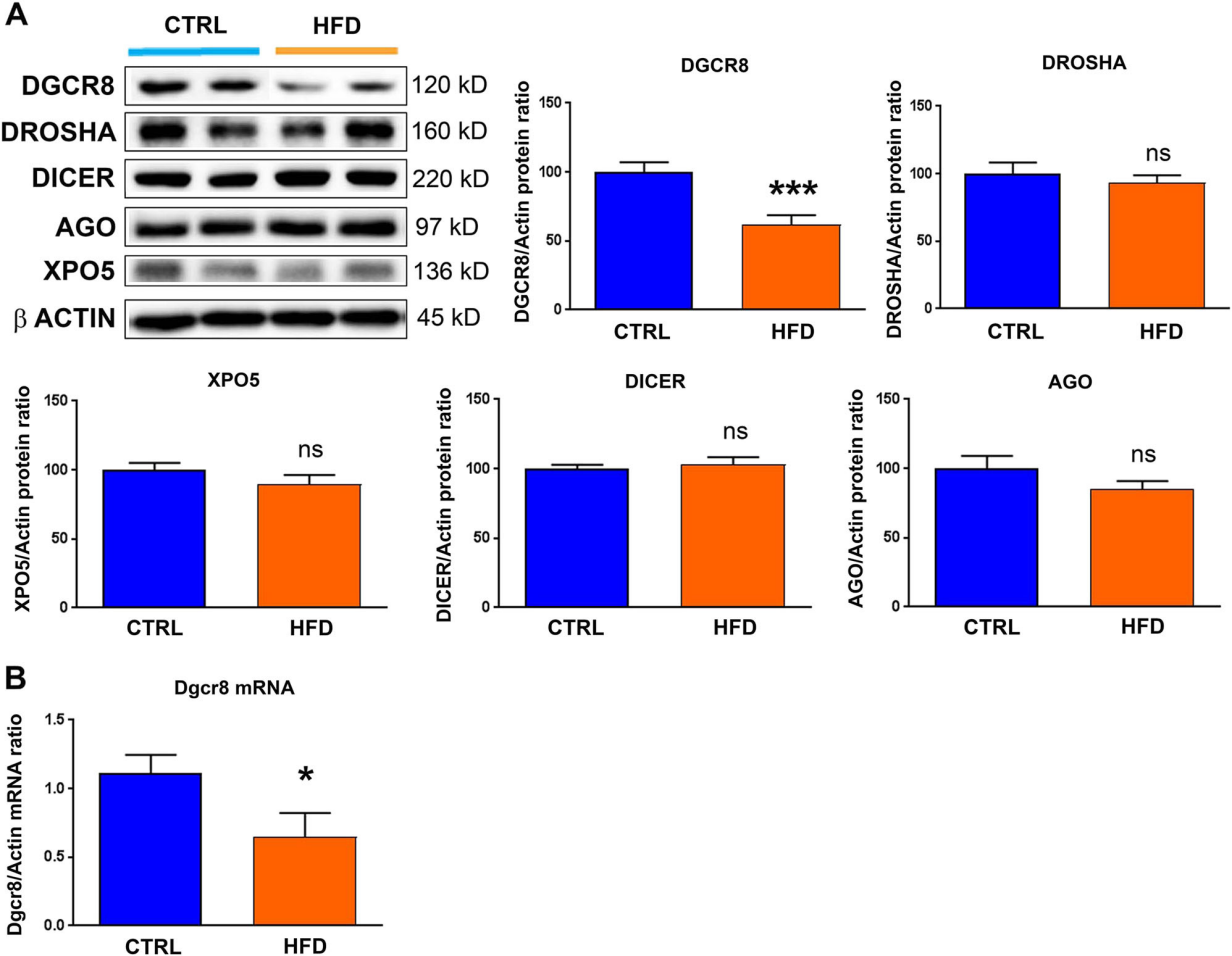
Effects of maternal exposure to high-fat diet on micro-RNA biogenesis. Protein levels of DGCR8, DROSHA, XPO5, DICER, and AGO2 (**a**) and mRNA levels of DGCR8 (**b**) were analyzed in the heart from male rat at post-natal day 77 under control chow diet (CTRL) or high-fat diet (HFD). (**a**) A representative immunoblot image is presented for each protein. Data are expressed as the mean ± SEM; *n* = 13 per group. *P < 0.05 and ***P < 0.001 compared to control, ns not statistically significant

Prompting a search for underlying mechanisms in micro-RNA down-regulation induced by maternal exposure to high-fat diet, we investigated the different steps of micro-RNA biogenesis by measuring the expression levels of key components of the micro-RNA biogenesis machinery: DROSHA, DGCR8, DICER, XPO5 and AGO2 (Fig. 6a). Among them, DGCR8 showed a significant decrease at the protein (Fig. 6a) and mRNA (Fig. 6b) levels when the animals were exposed to maternal high-fat diet. These data suggest that DGCR8-decreased expression may be responsible for micro-RNA down-regulation induced by maternal exposure to high-fat diet, and the subsequent cardiac alterations mediated by TGFβ pathway dysregulations.

### DGCR8 down-regulation effects on micro-RNA levels and TGFβ pathway

**Fig. 7:**
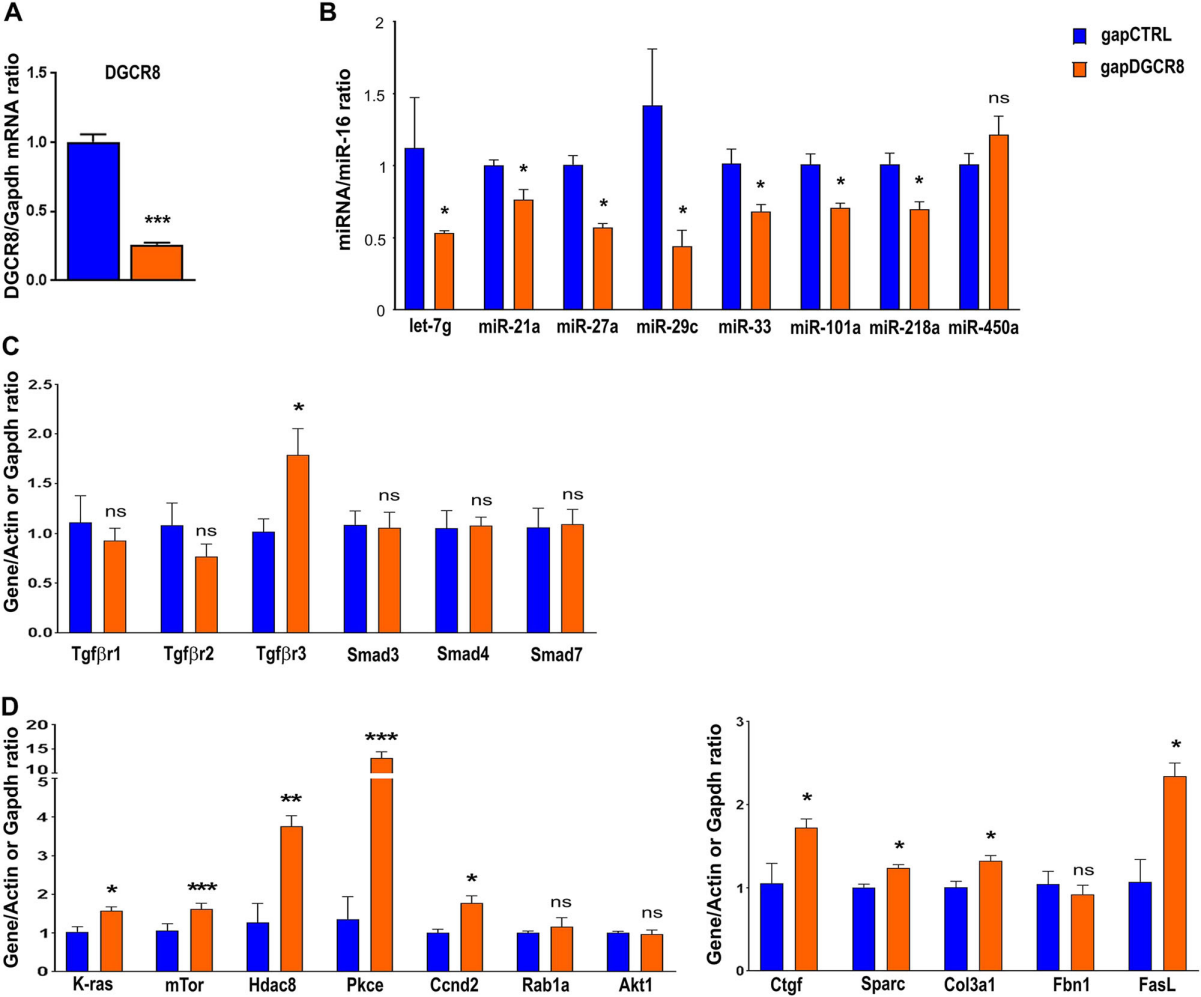
DiGeorge critical region 8 (DGCR8) down-regulation in cardiomyocytes alters the levels of micro-RNA and genes involved in transforming growth factor-beta (TGFβ)-mediated fibrosis and hypertrophy. H9C2 cells plated in 12-well plates were transfected with 20 pmol gapmeR control (negative control, gap CTRL) or gapmeR targeting DGCR8 (gap DGCR8) for 48 h. The results are representative of two independent experiments. In these cells, DGCR8 mRNA levels were measured by quantitative real-time PCR (RT-qPCR) (**a**). Let-7g, miR-21, miR-27a, miR-29c, miR-33, miR-101a, miR-218a and miR-450a levels were measured in cells transfected with gapmeRs (**b**). mRNA levels of putative target genes involved in TGFβ-mediated remodeling were analyzed (**c**) and (**d**). Data are expressed as the mean ± SEM. *P < 0.05, **P < 0.01 and ***P < 0.001 compared to control, ns not statistically significant

To delineate the role of DGCR8 in the regulation of micro-RNA levels in cardiomyocytes, we down-regulated its expression in H9C2 cell line. In these cells, the transfection of a specific gapmeR targeting *Dgcr8* (gap DGCR8) induced a 74% decrease in *Dgcr8* mRNA levels compared to the cells transfected with a control gapmeR (negative control, gap CTRL) (Fig. 7a). Difference in cell survival was not detected when Dgcr8 was inhibited (supplemental data). In contrast, the levels in let-7g, miR-21, miR-27a, miR-29c, miR-33, miR-101a and miR-218a were down-regulated in these cells (Fig. 7b). We then verified the role of *Dgcr8* and micro-RNA decrease on the regulation of genes involved in TGFβ-mediated fibrosis and hypertrophy pathway (Fig. 7b, c). We found that *Dgcr8* down-regulation through gapmeR transfection induced a significant increase in *Tgfβr3* (Fig. 7b). The mRNA levels of pro-hypertrophic genes such as *K-Ras, mTor, Hdac8, Prkce* and *Ccnd2* were upregulated when *Dgcr8* expression was decreased (Fig. 7c). Similarly, pro-fibrotic genes such as *Ctgf, Sparc, Col3a1* and *FasL* showed increased mRNA levels in cells where *Dgcr8* expression was inhibited (Fig. 7d). These data indicate that *Dgcr8* decrease in vitro can induce the down-regulation of a subset of micro-RNAs involved in TGFβ pathway regulation, through the regulation of multiple targets involved in the canonical and non-canonical pathways. This results in increased pro-hypertrophic and pro-fibrosis gene expression in cardiomyocytes. In view of these results, DGCR8 decrease appears as a potential origin for the cardiac fibrosis and hypertrophy driven by maternal exposure to high-fat diet.

### Hemin treatment rescues the levels of micro-RNAs and genes in cardiomyocytes with low levels of *Dgcr8*

**Fig. 8:**
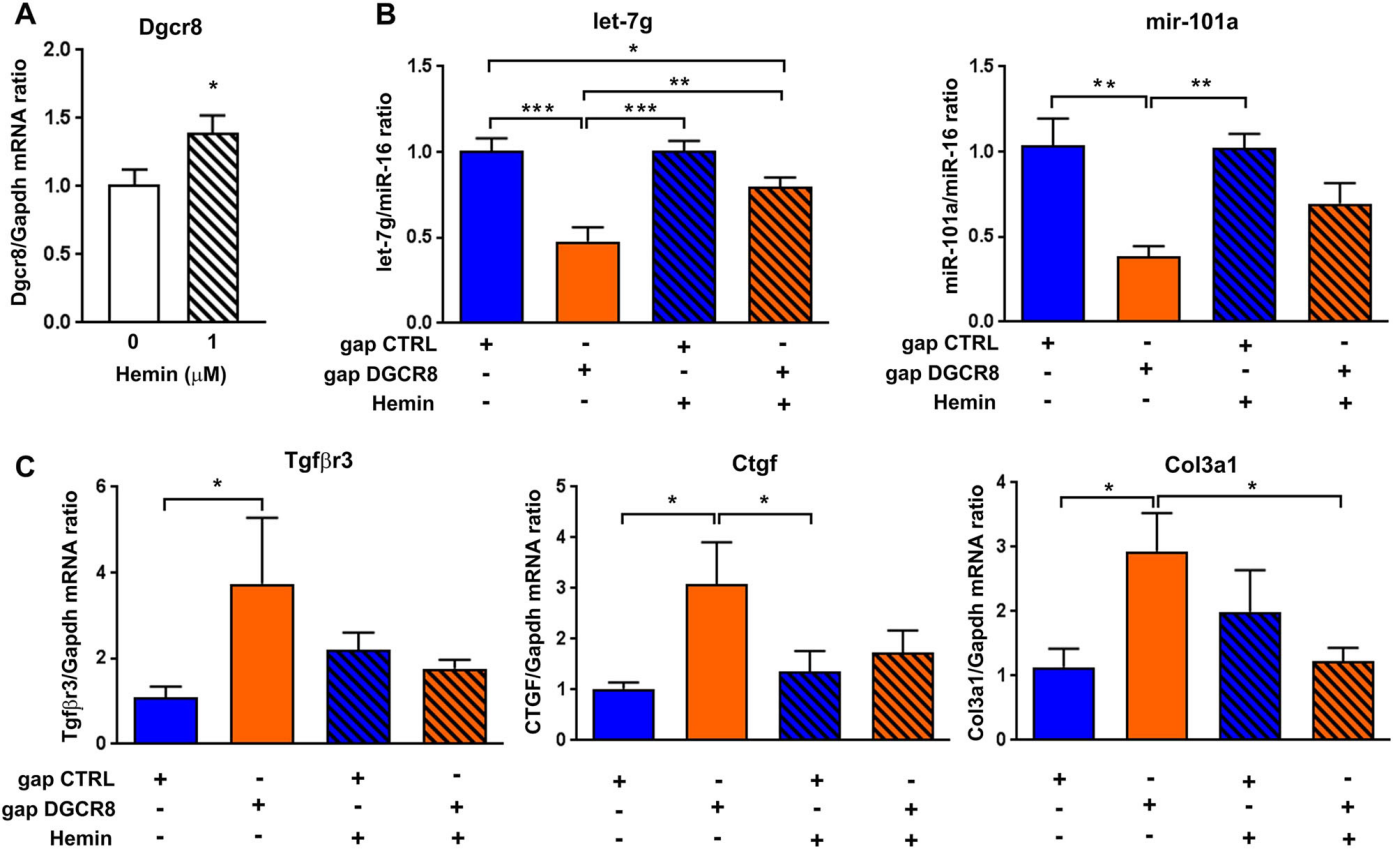
Hemin rescues the pro-fibrotic effect of DiGeorge critical region 8 (DGCR8) decrease in cardiomyocytes. (**a**) The effect of hemin on DGCR8 mRNA levels were measured in H9C2 cells. (**b**) miR-101a and (**c**) let-7g levels and the expression of transforming growth factor-beta (TGFβ)-mediated pro-fibrotic genes (*TgfβR3, Ctgf* and *Col3a1*) were measured in the cells transfected with the gapmeRs and treated with hemin (1 μM) or its vehicle (water, hemin 0 μM). Data are expressed as the mean ± SEM. *P < 0.05, **P < 0.01 and ***P < 0.001

Since DGCR8 down-regulation leads to alterations in cardiomyocytes, we wondered if we could rescue the phenotype by increasing DGCR8 expression and/or activity. Thus, we further tested the rescue of the phenotype through hemin treatment in cardiomyocytes where Dgcr8 expression was decreased. Hemin, which is a ferric form (Fe3+) of heme and which has been shown to stimulate the microprocessor activity in vitro^23–25^ and in vivo^26^, has been described as an efficient approach in the reversal of fibrosis in a model of hypertension^27^, and as a negative regulator of TGFβ pathway^28^. Therefore, we wondered if these beneficial effects of hemin on fibrosis and TGFβ pathway described in other models could be also found in cardiomyocytes, and if those effects are mediated by DGCR8 and micro-RNAs. To answer this question, cardiomyocytes were treated with 1 μM hemin. These cells exhibited increased *Dgcr8* mRNA levels compared to control cells (treated with vehicle) (Fig. 8a). Furthermore, in cells transfected with gapmeRs, the down-regulation in the anti-fibrotic micro-RNA miR-101a and let-7g levels induced by *Dgcr8* inhibition was reduced when the cells were treated with hemin (gap DGCR8 with hemin vs. gap CTRL) (Fig. 8b). Finally, the increase in pro-fibrotic genes induced by *Dgcr8* inhibition, including *Tgfβr3*, *Ctgf* and *Col3a1*, was not detected anymore when the cells were treated with hemin (gap DGCR8 with hemin vs. gap CTRL) (Fig. 8c). Overall, these data suggest that increasing DGCR8 expression and/or activity in cardiomyocytes with hemin tends to rescue the pro-fibrotic phenotype mediated by TGFβ pathway.

## Discussion

Our study addresses critical health problems (obesity and heart diseases) associated with modern lifestyle diet (high-fat diet). Maternal obesity and high-fat diet have been associated with increased risk for heart diseases, notably through the induction of cardiac remodeling. Indeed, maternal exposure to high-fat diet induces cardiac hypertrophy and fibrosis that are observed early during development (at PND1 and PND10)^6,29^. Our current study showed that maternal exposure to high-fat diet induced long-term histological and molecular changes in the heart prior to the onset of overt obesity. Notably, the present work highlighted the decrease in a micro-RNA subset as a potential mechanistic basis linking maternal nutritional imbalance early during development and mortality related to cardiac diseases. Here, the down-regulation of a subset of cardiac micro-RNAs induced by maternal exposure to high-fat diet for 35 days, during pregnancy and lactation, was detected later during life, in adulthood. Importantly, this observation indicates that a transient exposure to dietary distress during developmental sensitive periods induces sustained down-regulation of a micro-RNA subset. This observation is reminiscent of the professor Barker hypothesis related to the concept of the developmental origins of health and disease. In this context, it is noteworthy that altered micro-RNA patterns may be associated with disturbed developmental processes during fetal or neonatal life. Here, our work further highlighted micro-RNA alterations as a possible cause for TGFβ-mediated cardiac fibrosis and hypertrophy induced by early developmental exposure to dietary challenge. Various intracellular signaling pathways are thought to play a critical role in the pathological remodeling of the heart and, importantly, are activated both independently and downstream of TGF-β. Among the targets we identified in this work, *Tgfβr3* was up-regulated. TGFβRIII is a widely expressed accessory receptor^30^ for multiple classes of growth factors (including TGF-βs, along with fibroblast growth factor (FGF), inhibin, activin, bone morphogenetic protein (BMP)-2, BMP-4 and BMP-7), and as such, TGFβRIII has the potential to influence diverse cellular processes. TGFβRIII enhances TGFβ signaling^31,32^ and also activates RAS-ERK and p38 MAPK in a ligand-independent manner, both involved in fibrosis^33^ and hypertrophy, in a SMAD-independent manner. Importantly, we newly identified alterations in DGCR8 expression as a potential cause for long-term cardiac micro-RNA down-regulation in this model. DGCR8 importance in micro-RNA biogenesis and in pathophysiology has been revealed in human 22q11.2 deletion syndrome (known as DiGeorge syndrome)^34^ and in animal model where the Dgcr8 gene was heterozygously deleted^35^. The haploinsufficiency of Dgcr8 reduces the abundance of DGCR8 protein, resulting in lower pri-micro-RNA processing efficiency and abnormal expression of a subset of micro-RNAs^36^. In humans, the DiGeorge syndrome is associated with increased risk for congenital heart defects^37^. In mice, cardiomyocyte-specific deletion of *Dgcr8* induces cardiac fibrosis with left ventricular malfunction progressing to a dilated cardiomyopathy and premature lethality^12^. Our data highlighted a new potential pathway through which DGCR8 alterations could lead to cardiac hypertrophy and fibrosis involving at least in part TGFβ pathway. Although modulation of TGF-β signaling with different approaches has proven effective pre-clinically and clinically in the treatment of multiple pathologies^38–40^, the rescue of the phenotype remains partial. In the light of our findings, DGCR8 appears as an attractive candidate in the reversal of the cardiac phenotype induced by early exposure to dietary challenge since its modulation might impact the TGFβ signaling pathway at multiple levels. In this sense, studies have demonstrated the possibility of regulating DGCR8 expression and activity in several ways including by cofactor binding, phosphorylation, acetylation and proteolytic cleavage^26,41–43^. Among them, hemin, a ferric form (Fe3+) of heme, has proven its efficiency in the up-regulation of DGCR8 activity. Hemin is incorporated in a DGCR8 dimer and stimulates the microprocessor activity in vitro^23–25^ and in vivo^26^. Mechanistically, DGCR8 was shown to depend on hemin to recognize the apical loop of primary micro-RNA^44^. Furthermore, the hemin level in human cells can impact the expression of several micro-RNAs^26^ and decrease cardiac oxidative stress and fibrosis in a rat model of systemic hypertension^27^. Here, we showed that hemin can restore micro-RNA levels in cardiomyocytes while *Dgcr8* expression was decreased, supporting the physiological function of hemin in stimulating micro-RNA biogenesis and benefits for heart functions. Similarly, through the stable binding to DGCR8, Cobalt (III) protoporphyrin has been shown to compensate micro-RNA processing deficiency and restore micro-RNA levels in models where DGCR8 expression was decreased in vivo^45^. Thus, it is of high interest to test the potential of hemin treatment in the prevention and/or reversal of cardiac alterations in animals exposed to dietary challenge early during development.

More widely, alterations in primary micro-RNA processing have been reported in other models of exposure to environmental challenges such as cigarette smoke^46^, and also in aging^47^ and in a number of human diseases including cancer^48^, schizophrenia^49^, kidney^50^ and auto-immune diseases^51^. To restore micro-RNA processing by enhancing the activity of DGCR8 protein using small molecule agents represents an interesting candidate. Thus, the targeting of DGCR8 offers opportunities for intervention and may emerge as an exciting and important arena for drug development. Also, micro-RNAs are strikingly stable and can be detected in body fluids, including blood. This observation has raised the possibility that micro-RNAs may be probed in the circulation and can serve as novel diagnostic markers^52,53^. Thus, this is of high interest to evaluate the potential of the micro-RNAs identified in our study as early blood-based biomarkers of cardiac alterations induced by exposure to dietary challenges. Our micro-RNA profile could provide a novel strategy for diagnosis in chronic cardiac dysfunctions programmed by environmental stressors early in life.

## Methods

### Materials

Protease inhibitor cocktail, Tween-20, Hemin, High Pure miRNA Isolation Kit and other reagents were purchased from Sigma Aldrich. Antibodies raised against DGCR8, β-actin, anti-rabbit and anti-mouse IgG HRP-linked antibody; BCA Protein Assay Kit; RIPA buffer; phosphatase inhibitor cocktail; chemiluminescent western pico plus chemiluminescent substrate, Power up SybrGreen Master Mix, High Capacity Reverse Transcriptions Kit, TaqMan Advanced miRNA cDNA Synthesis Kit and miRNA Advanced Taqman assays were purchased from Thermo Fisher Scientific, Inc. The polyvinylidene difluoride (PVDF) membranes were obtained from Amersham. DICER, DROSHA, XPO5, CTGF, SPARC and GAPDH antibodies were obtained from Santa Cruz Biotechnology. Antibodies raised against phospho-p70 S6K and p70 S6K were purchased from Cell Signaling Technology, Inc. GapmeRs were purchased from Qiagen.

### In vivo experimental studies

The animal experiments were performed in accordance with EU legislation (Directive 2010/63/EU) and approved by a local animal care and use committee (Comité Institutionnel d’Éthique Pour l’Animal de Laboratoire-CIEPAL-Azur-Agreement number: NCE-2013-109; Protocol No. 2015-62). Pregnant Sprague Dawley rats at gestational day 7 (GD7) (Janvier, Le Genest Saint Isle, France) were individually housed in temperature-controlled rooms with 12 h light/12 h dark cycles and ad libitum access to water and feed. Upon arrival, pregnant females were either fed with chow diet (R03, Safe, Augy, France, *n* = 5) or high-fat diet (SNIFF 60%, SSNIFF, Soest, Germany, so-called HFD, *n* = 5). At birth, pups were sexed by anogenital distance and litters were culled to 5 pups per dam by removing or adding female pups as necessary. The remaining pups were killed by CO_2_ inhalation. At PND21, all male rats were given a chow (R03). Animals were killed by CO_2_ inhalation on PND77 (young adults). Each group contains 18 male rats.

### Heart sampling

The heart was quickly harvested and immediately frozen in liquid nitrogen or fixed in paraformaldehyde and paraffin embedded. Frozen tissues were grounded into fine powder for further molecular analyses.

### Histology, immunohistochemistry and immunofluorescence

Heart sections (5 μm) were stained with Masson’s trichrome. Five animals per group were analyzed. Image acquisition was performed on a Nikon Eclipse Ti microscope. Fibrotic area quantification was performed as described by Chen et al.^54^ with ImageJ software.

### Cell culture and transfection

H9C2 cardiomyocytes cell line was maintained in medium supplemented with 10% heat-inactivated fetal bovine serum at 37 °C in a humidified, CO_2_-controlled (5%) incubator. Cells were transfected with 20 pmol gapmeR targeting *Dgcr8* (gap DGCR8) or a negative control using Lipofectamine RNAimax Transfection reagent, according to the manufacturer’s protocol. At 48 h following transfection, cells were harvested for RNAs and protein extraction. For rescue experiments, 24 h after transfection, cells were incubated with 1 μM hemin, or with the vehicle (water) for 48 h.

### Cell survival assay

Cells were seeded on 96-well plate and transfected with gapmeRs. Cells were washed with phosphate buffer saline, fixed in ice-cold methanol for 10 min and stained with 0.5% crystal violet solution at room temperature for 10 min. Optical density was measured on a plate reader Infinite M200Pro (Nanoquant).

### Protein extraction

Aliquots (~20 mg) of heart tissue powder were homogenized in RIPA buffer (supplemented with 1% proteases and phosphatases inhibitor cocktail). Protein concentration was determined with the bicinchoninic acid assay. In total, 10 to 30 μg were used for western blot analysis.

### Western blotting analysis

Lysates were separated using sodium dodecyl sulfate–polyacrylamide gel electrophoresis (SDS-PAGE) and transferred to PVDF membranes. The membranes were blocked with 1% bovine serum albumin in phosphate-buffered saline (pH 7.4) containing 0.05% Tween-20 and were incubated with primary antibodies and horseradish peroxidase (HRP)-conjugated secondary anti-rabbit or -mouse antibodies. The proteins of interest were visualized using SuperSignal West Pico PLUS Chemiluminescent Substrate. Membranes were scanned with a luminescent image analyzer camera G:Box (Syngene) and quantified with Gene Tools software (Syngene).

### RNA isolation

Total RNA was isolated from frozen heart powder using 1 ml TRIzol reagent and 200 μl chloroform. The aqueous phase was precipitated with 1.5 vol of ethanol 100% at 4 °C overnight followed by 75% ethanol. Total RNAs were further purified on a column. The RNA purity was checked by optical density measurement at 230, 260 and 280 nm. The quantities of total RNAs and micro-RNAs were evaluated with fluorescent-based methods on a Qubit apparatus (Life technologies, Inc.). RNA integrity was evaluated with a Fragment Analyzer (Agilent).

### MIRNA microarray experiment

Sample quality was tested and was ranging from RQN (RNA Quality Number) 6.7 to RQN 8.0.

To assess the effect of maternal exposure to high-fat diet on cardiac miRNA levels, RNAs from four biological replicates of high-fat diet vs. control diet were hybridized on the microarrays (SurePrint Rat miRNA Microarrays, Agilent). Each sample was prepared according to the Agilent’s micro-RNA Microarray System protocol. The arrays were scanned with an Agilent microarray scanner using high dynamic range settings as specified by the manufacturer. Agilent Feature Extraction Software Version 10.7.3.1 was used to extract the data. Normalization was performed on the total Gene Signal from Agilent “GeneView” data files in R language, version 3.4, an open source statistical scripting language (http://www.r-project.org). Invariant-based normalization was performed as previously described^55^. After normalization, micro-RNA not detected in any sample were filtered, leaving 240 miRNAs for analysis. For statistical analyses, we performed linear model using Bioconductor package Limma with the two conditions (chow diet and high-fat diet) as factors, extraction of contrasts of interest, moderated *t*-test for comparisons were applied, adjustment of *P* values by the Benjamini–Hochberg method and controlling for false discovery rate were realized.

### Pathway analysis

We performed computational target prediction analysis coupled to pathway analysis using DIANA-miRPath (v3.0) software. Potential target RNAs for down-regulated micro-RNAs from each cluster were identified using the prediction algorithm DIANA-microT-CDS (v5.0). The potential mRNA targets for the down-regulated micro-RNAs were then subjected to KEGG pathway analysis. The DIANA-miRPath software calculates the significance for all the miRNA–mRNA pairs in a pathway, and then combines them into a merged *P* value for each pathway^21^.

### Real-time quantitative PCR for mRNA expression

In total, 200 ng of RNAs was reverse transcribed using High Capacity Reverse Transcription Kit according to the manufacturer’s protocols in a SimplyAmp Thermal Cycler (Life Technologies, Inc.). RT-qPCR was performed with 2 μl of diluted cDNA (1:20) using the I-Taq Universal SybrGreen Master Mix and both sense and antisense primers in a final volume of 10 μl in a Quantstudio 3 apparatus (Life Technologies, Inc.). The primers that were used are listed in supplemental table S1. Data were normalized to* β-actin* or to *Gapdh* using the Ct method.

### Micro-RNA expression quantification by RT-qPCR

In total, 10 ng of RNAs was reverse transcribed with advanced miRNA RT kit on a SimplyAmp Thermal Cycler (Life Technologies, Inc.). RT product was diluted by 10-fold, and 2.5 µl was used for qPCR analyses. qPCR was performed with TaqMan Advanced Master Mix according to the manufacturer’s protocol on a Quantstudio 3 apparatus (Life Technologies, Inc.). The relative expression levels of miRNAs were calculated using the comparative ΔΔCt method by normalizing to miR-16 levels.

### Data analysis

The data from the different experiments were analyzed with GraphPad Prism software version 6.05 (GraphPad Software, Inc.). The values were expressed as mean ± SEM to account for sample and animal variation within a dataset. Student’s *t-*test was performed to determine whether there were differences between two groups, and analysis of variance when multiple groups were compared. *P* < 0.05 was considered statistically significant.

## Supplementary information

supplementary information
