# Supplementary material for: Long-term impact of maternal high-fat diet on offspring cardiac health: role of micro-RNA biogenesis
Source: Cell Death Discov. 2019 Mar 1;5:71. doi: 10.1038/s41420-019-0153-y (PMC6397280; doi:10.1038/s41420-019-0153-y)
Supplement: Supplementary file 1 — supplementary information [file 41420_2019_153_MOESM1_ESM.pdf]

FIGURE S1

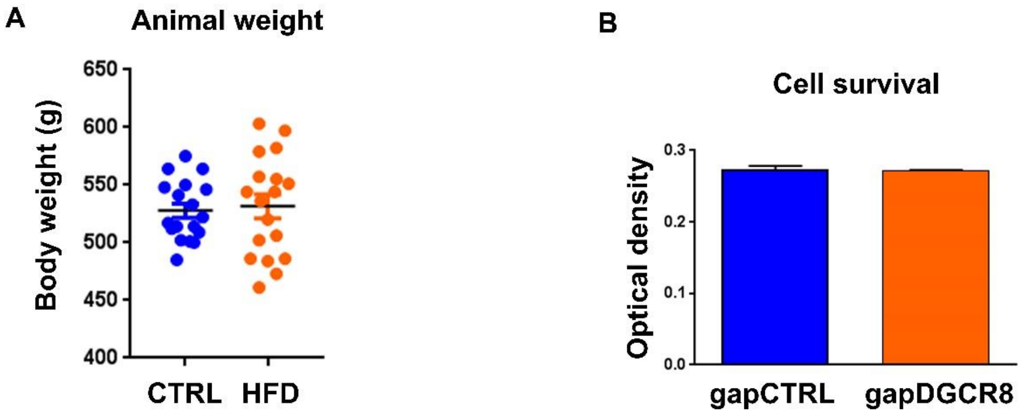

A
Animal weight
Body weight (g)
650
600
550
500
450
400
CTRL
HFD
B
Cell survival
Optical density
0.3
0.2
0.1
0.0
gapCTRL
gapDGCR8

FIGURE S2

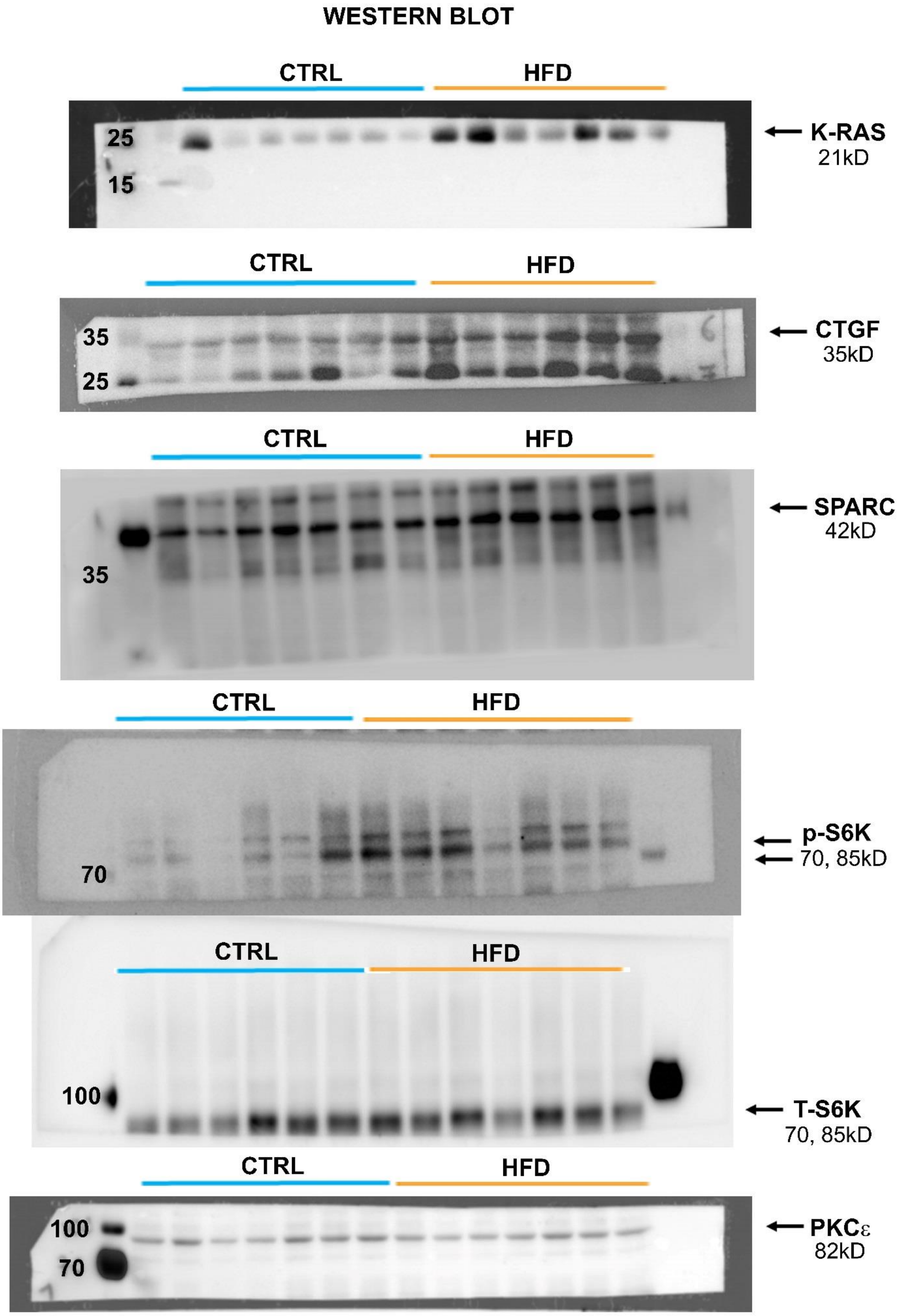
WESTERN BLOT
CTRL
HFD
25
15
K-RAS
21kD
CTRL
HFD
35
25
CTGF
35kD
CTRL
HFD
35
SPARC
42kD
CTRL
HFD
70
p-S6K
70, 85kD
CTRL
HFD
100
T-S6K
70, 85kD
CTRL
HFD
100
70
PKCε
82kD

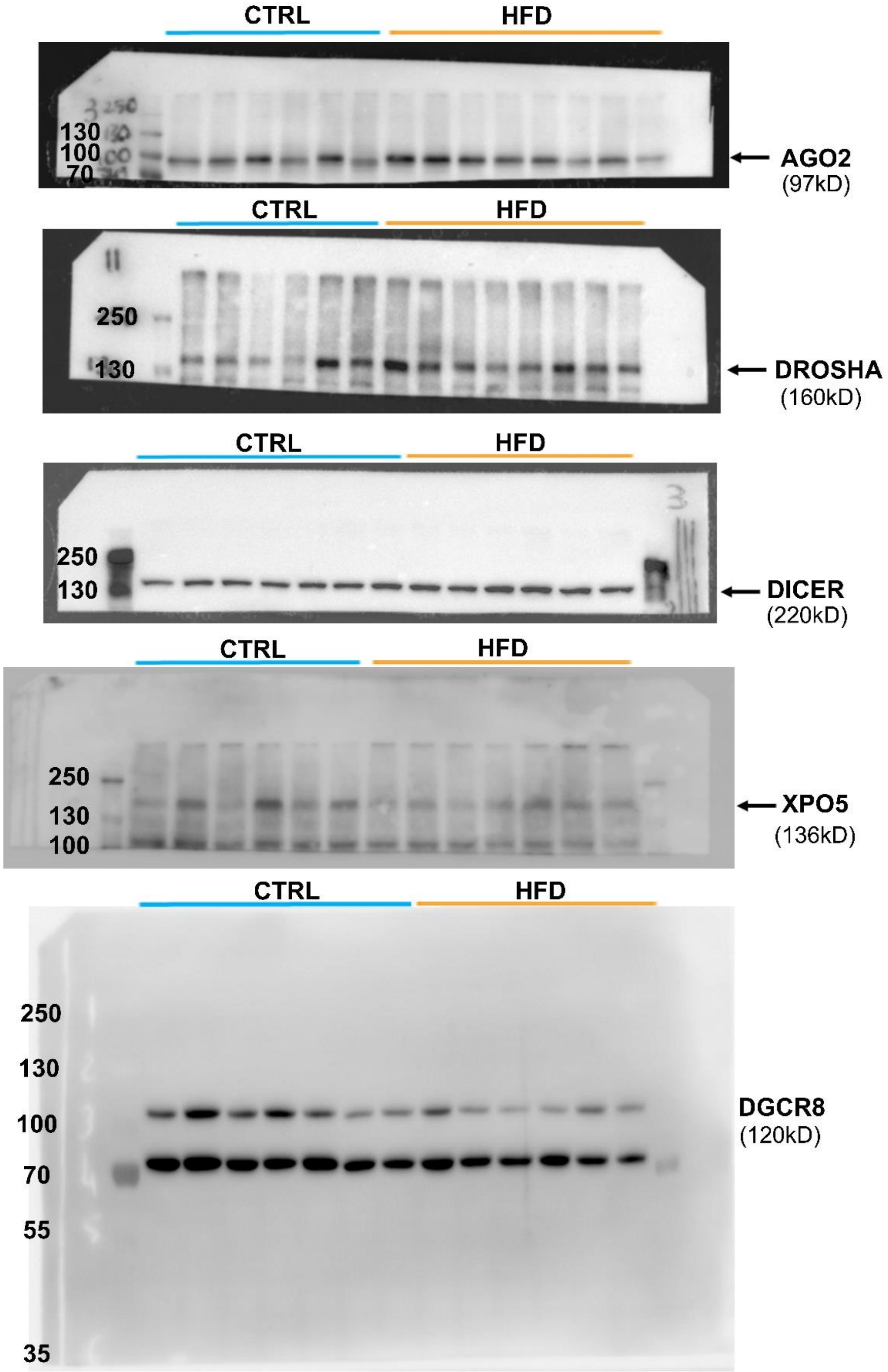
CTRL
HFD
130
100
70
AGO2
(97kD)
CTRL
HFD
250
130
DROSHA
(160kD)
CTRL
HFD
250
130
DICER
(220kD)
CTRL
HFD
250
130
100
XPO5
(136kD)
CTRL
HFD
250
130
100
70
55
35
DGCR8
(120kD)

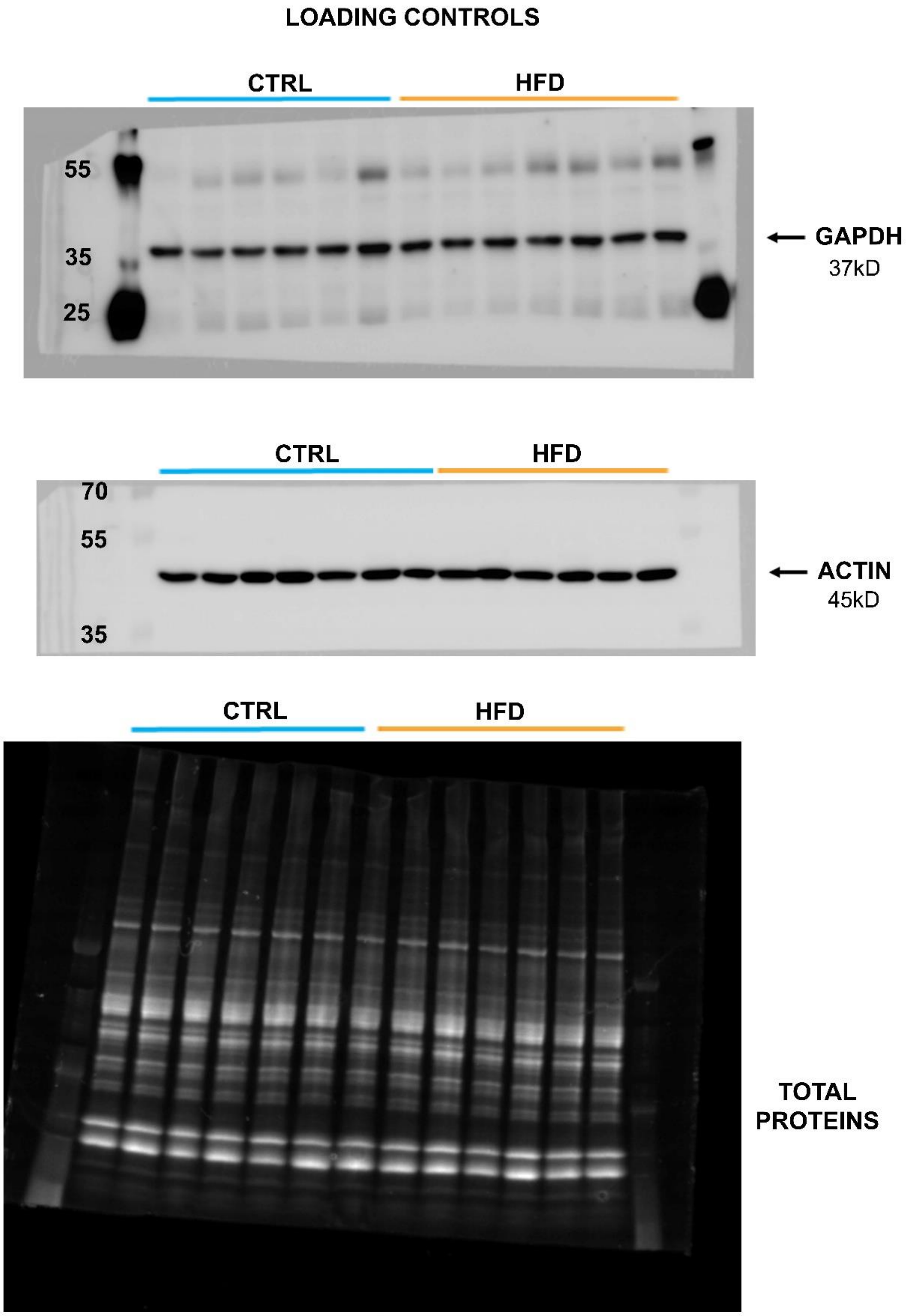
LOADING CONTROLS
CTRL
HFD
55
35
25
GAPDH
37kD
CTRL
HFD
70
55
35
ACTIN
45kD
CTRL
HFD
TOTAL
PROTEINS

FIGURE S3

A

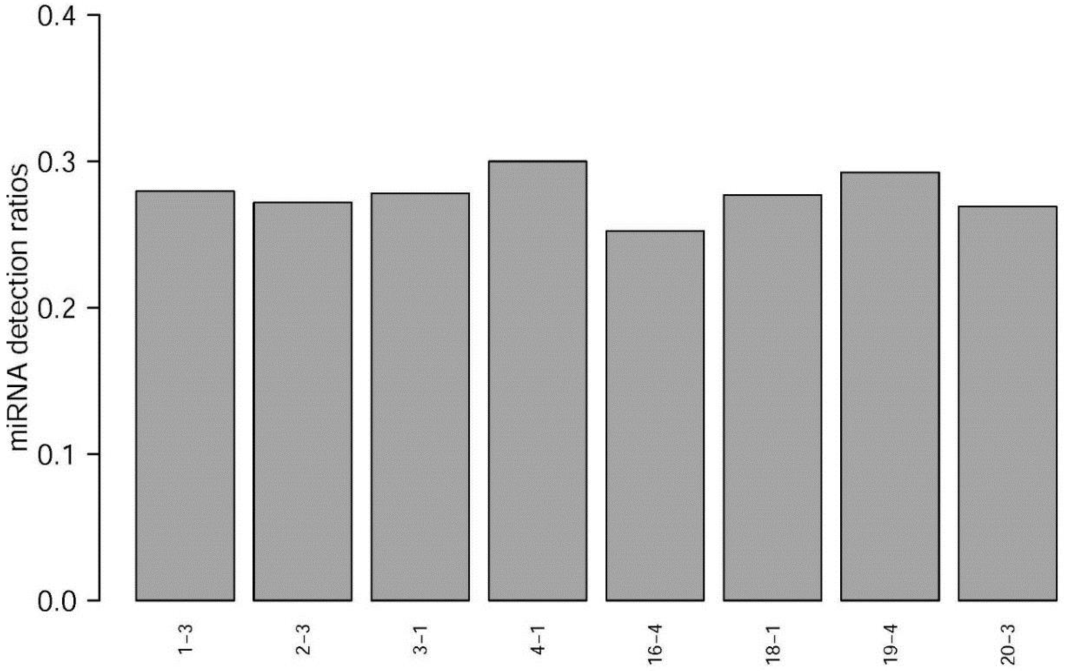

0.4
0.3
0.2
0.1
0.0
miRNA detection ratios
1−3
2−3
3−1
4−1
16−4
18−1
19−4
20−3

B

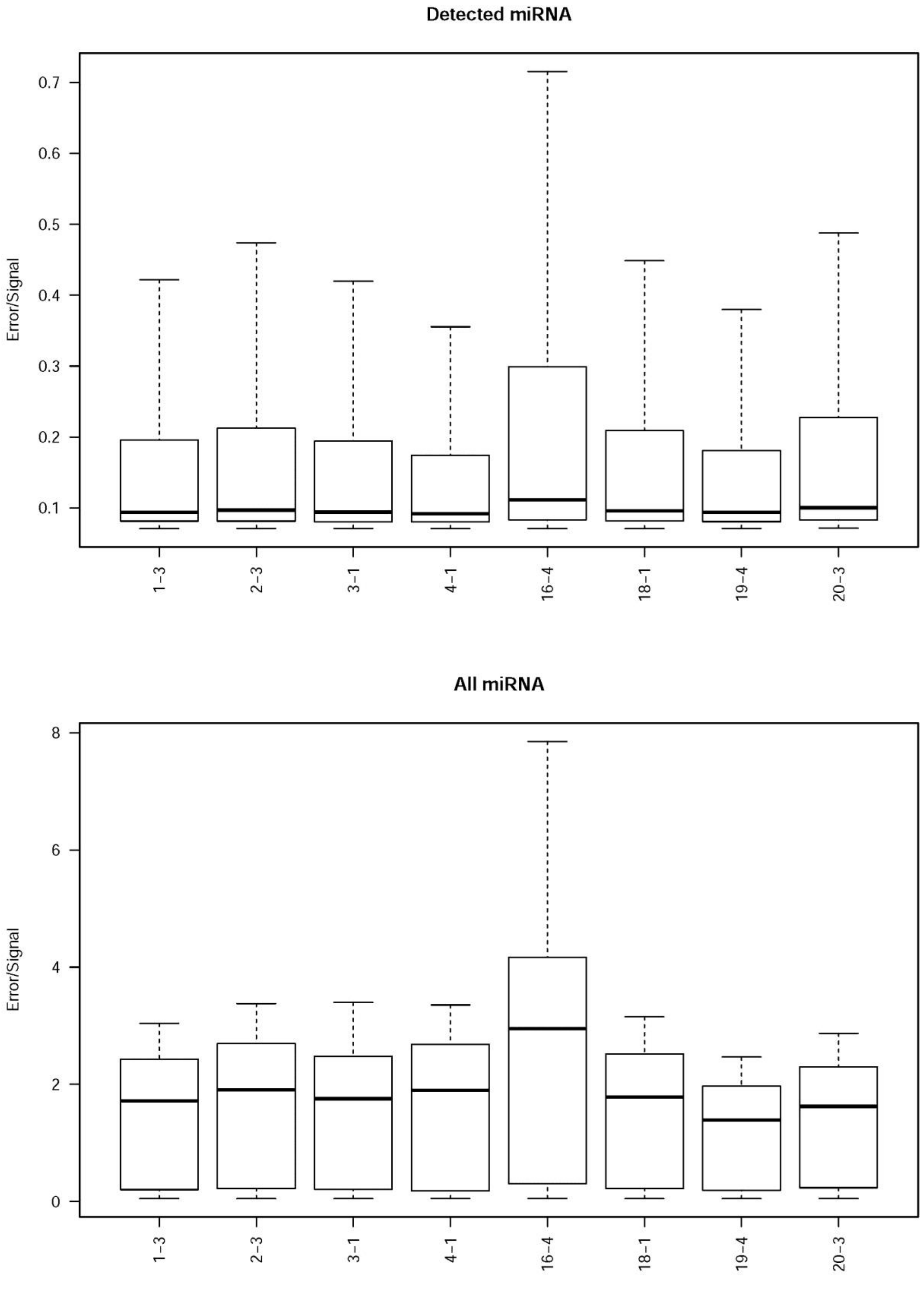
Detected miRNA
Error/Signal
0.1
0.2
0.3
0.4
0.5
0.6
0.7
1−3
2−3
3−1
4−1
16−4
18−1
19−4
20−3
All miRNA
Error/Signal
0
2
4
6
8
1−3
2−3
3−1
4−1
16−4
18−1
19−4
20−3

C

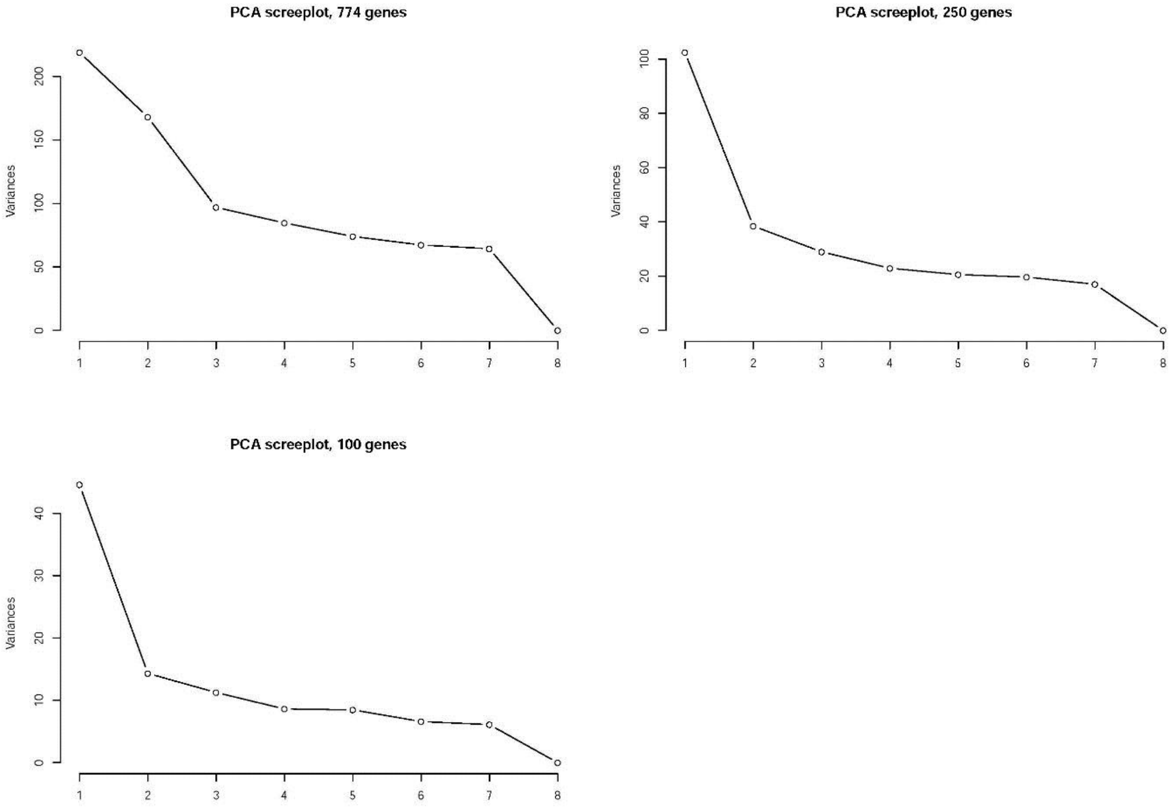
PCA screeplot, 774 genes
PCA screeplot, 250 genes
PCA screeplot, 100 genes
Variances
0
50
100
150
200
20
40
60
80
10
30
1
2
3
4
5
6
7
8

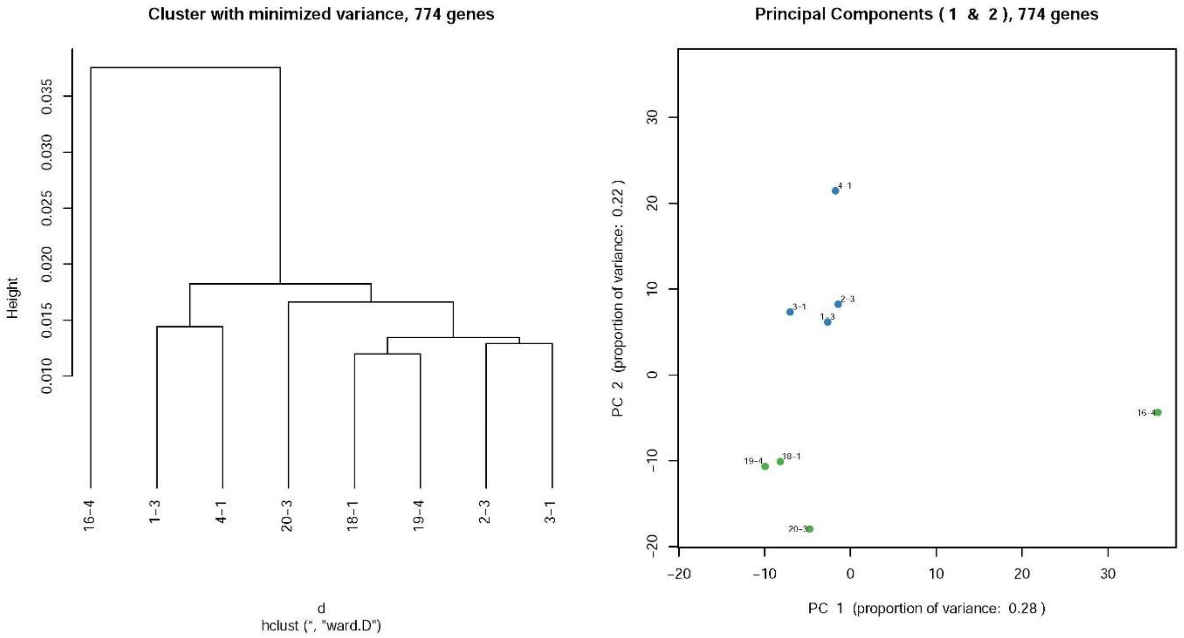
Cluster with minimized variance, 774 genes
Height
0.010
0.015
0.020
0.025
0.030
0.035
16−4
1−3
4−1
20−3
18−1
19−4
2−3
3−1
d
hclust (*, "ward.D")
Principal Components (1 & 2), 774 genes
PC 2 (proportion of variance: 0.22)
PC 1 (proportion of variance: 0.28)
−20
−10
0
10
20
30
4-1
3-1
2-3
1-3
16-4
19-4
18-1
20-3

54

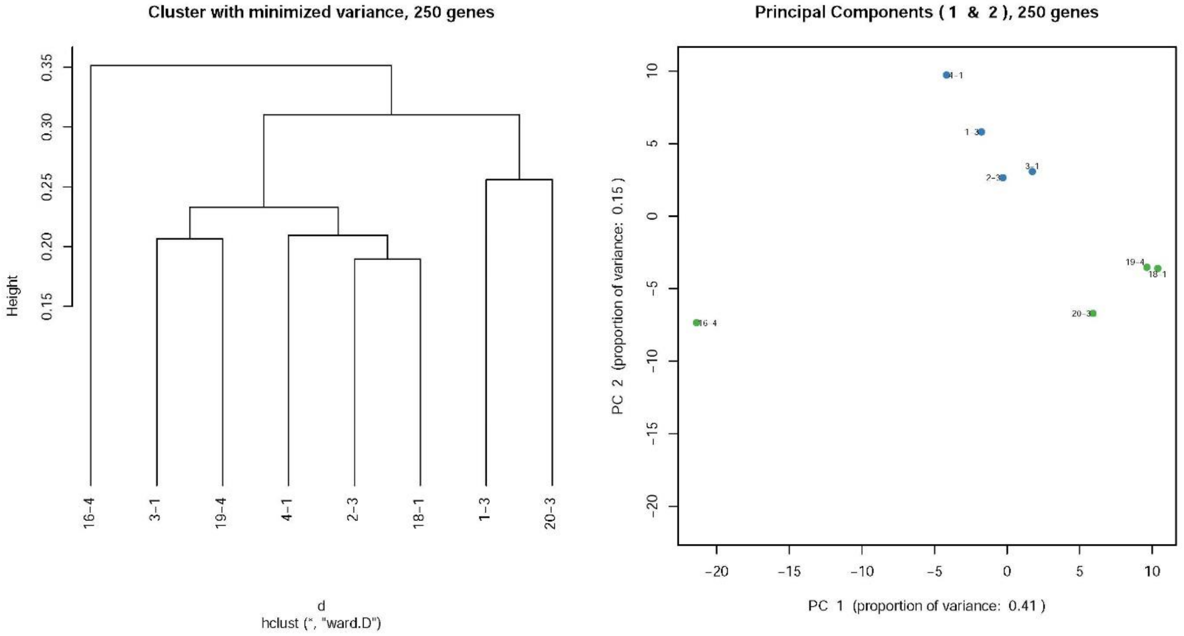
Cluster with minimized variance, 250 genes
Height
0.15
0.20
0.25
0.30
0.35
16−4
3−1
19−4
4−1
2−3
18−1
1−3
20−3
d
hclust (*, "ward.D")
Principal Components (1 & 2), 250 genes
PC 2 (proportion of variance: 0.15)
PC 1 (proportion of variance: 0.41)
−20
−15
−10
−5
0
5
10
4-1
1-3
3-1
2-3
19-4
18-1
20-3
16-4

55

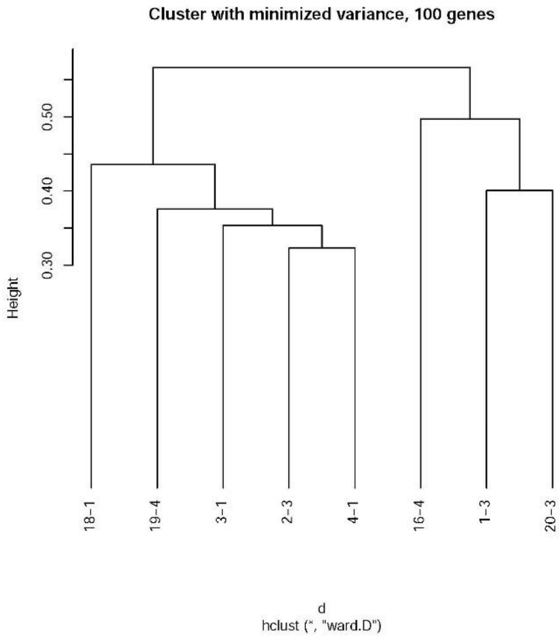
Cluster with minimized variance, 100 genes
Height
0.30
0.40
0.50
18−1
19−4
3−1
2−3
4−1
16−4
1−3
20−3
d
hclust (*, "ward.D")

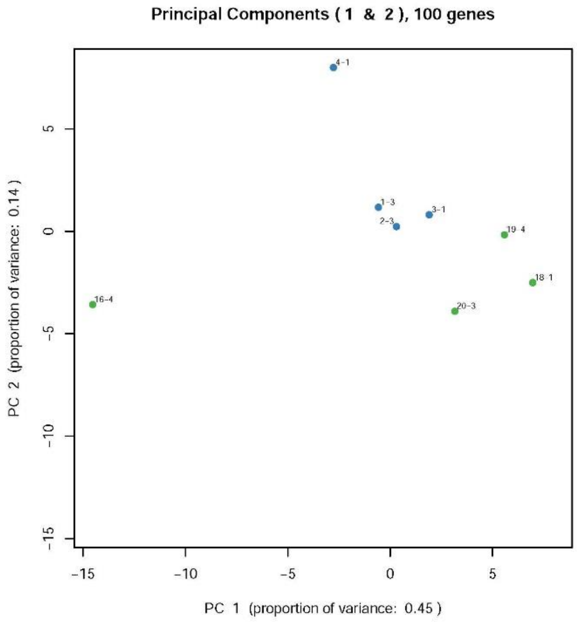
Principal Components (1 & 2), 100 genes
PC 2 (proportion of variance: 0.14)
PC 1 (proportion of variance: 0.45)
4−1
1−3
2−3
3−1
19−4
18−1
20−3
16−4
−15
−10
−5
0
5

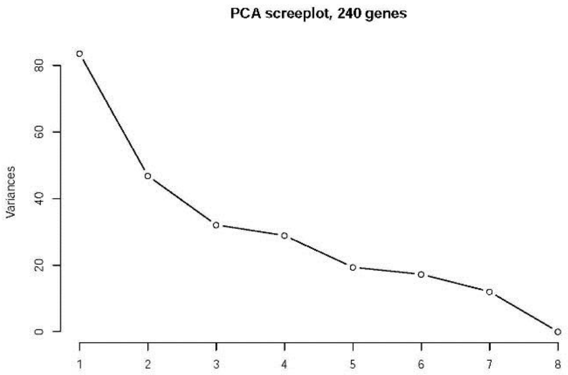
PCA screeplot, 240 genes
Variances
0
20
40
60
80
1
2
3
4
5
6
7
8

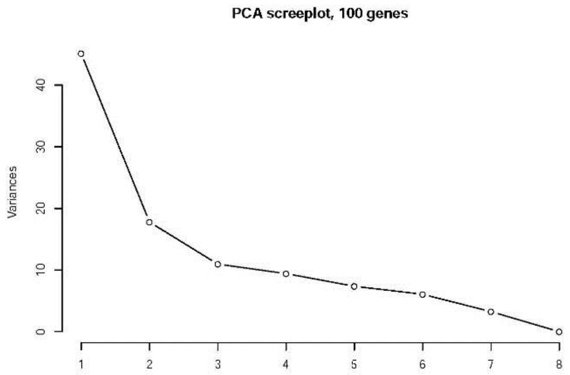
PCA screeplot, 100 genes
Variances
0
10
20
30
40
1
2
3
4
5
6
7
8

D

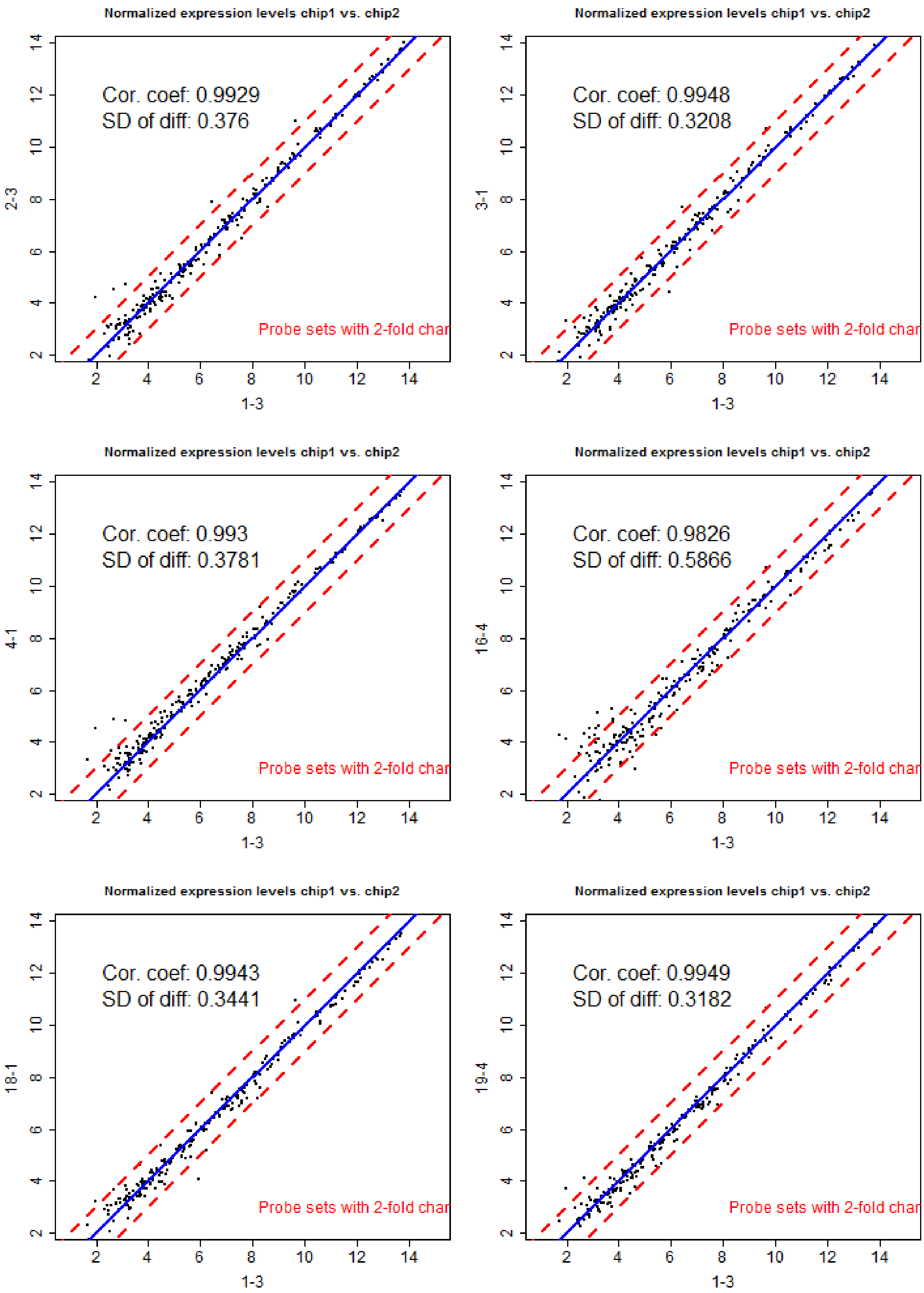
Normalized expression levels chip1 vs. chip2
Cor. coef: 0.9929
SD of diff: 0.376
Probe sets with 2-fold char
2-3
1-3
Normalized expression levels chip1 vs. chip2
Cor. coef: 0.9948
SD of diff: 0.3208
Probe sets with 2-fold char
3-1
1-3
Normalized expression levels chip1 vs. chip2
Cor. coef: 0.993
SD of diff: 0.3781
Probe sets with 2-fold char
4-1
1-3
Normalized expression levels chip1 vs. chip2
Cor. coef: 0.9826
SD of diff: 0.5866
Probe sets with 2-fold char
16-4
1-3
Normalized expression levels chip1 vs. chip2
Cor. coef: 0.9943
SD of diff: 0.3441
Probe sets with 2-fold char
18-1
1-3
Normalized expression levels chip1 vs. chip2
Cor. coef: 0.9949
SD of diff: 0.3182
Probe sets with 2-fold char
19-4
1-3

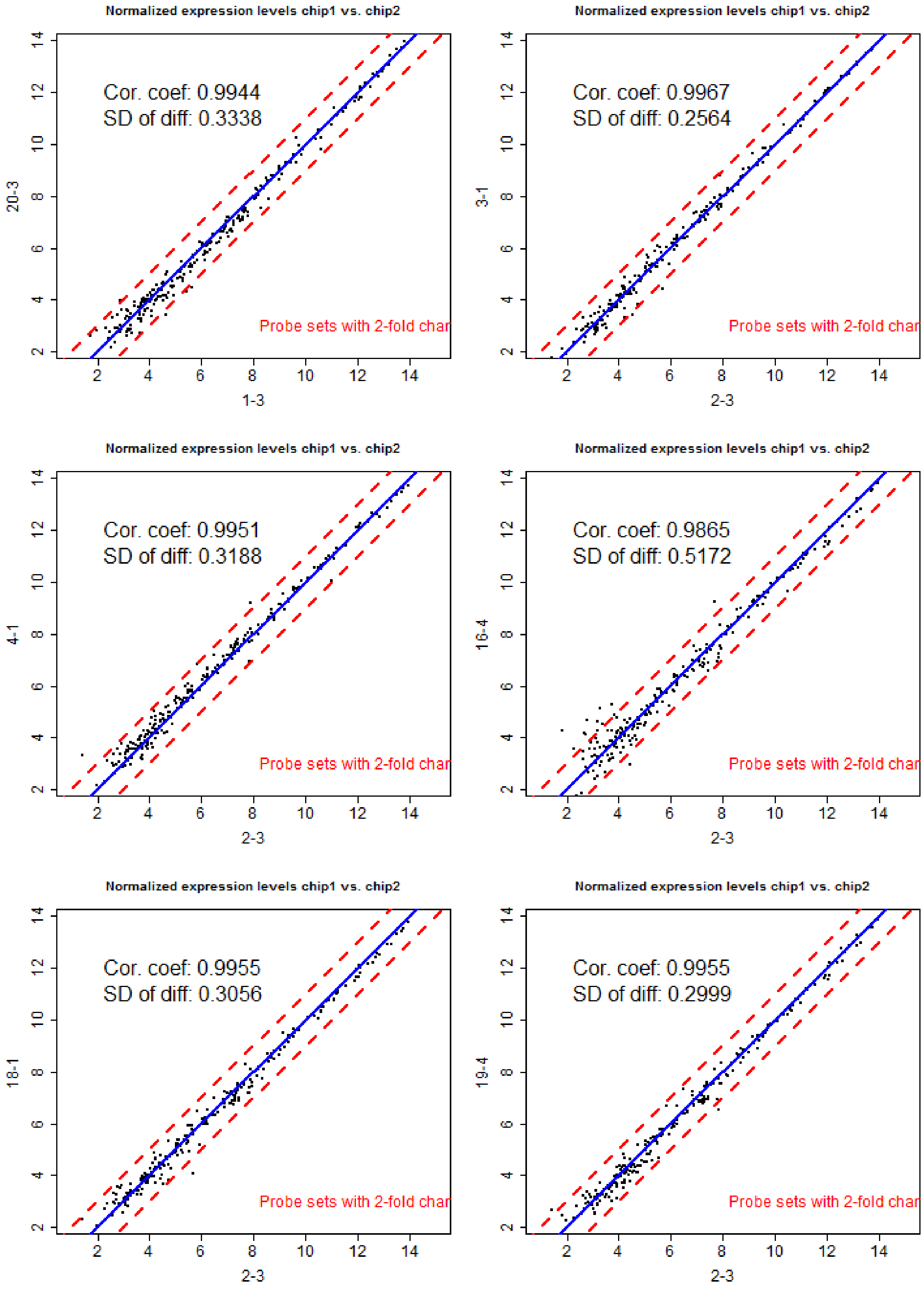

Normalized expression levels chip1 vs. chip2
Cor. coef: 0.9944
SD of diff: 0.3338
Probe sets with 2-fold char
20-3
1-3
Normalized expression levels chip1 vs. chip2
Cor. coef: 0.9967
SD of diff: 0.2564
Probe sets with 2-fold char
3-1
2-3
Normalized expression levels chip1 vs. chip2
Cor. coef: 0.9951
SD of diff: 0.3188
Probe sets with 2-fold char
4-1
2-3
Normalized expression levels chip1 vs. chip2
Cor. coef: 0.9865
SD of diff: 0.5172
Probe sets with 2-fold char
16-4
2-3
Normalized expression levels chip1 vs. chip2
Cor. coef: 0.9955
SD of diff: 0.3056
Probe sets with 2-fold char
18-1
2-3
Normalized expression levels chip1 vs. chip2
Cor. coef: 0.9955
SD of diff: 0.2999
Probe sets with 2-fold char
19-4
2-3

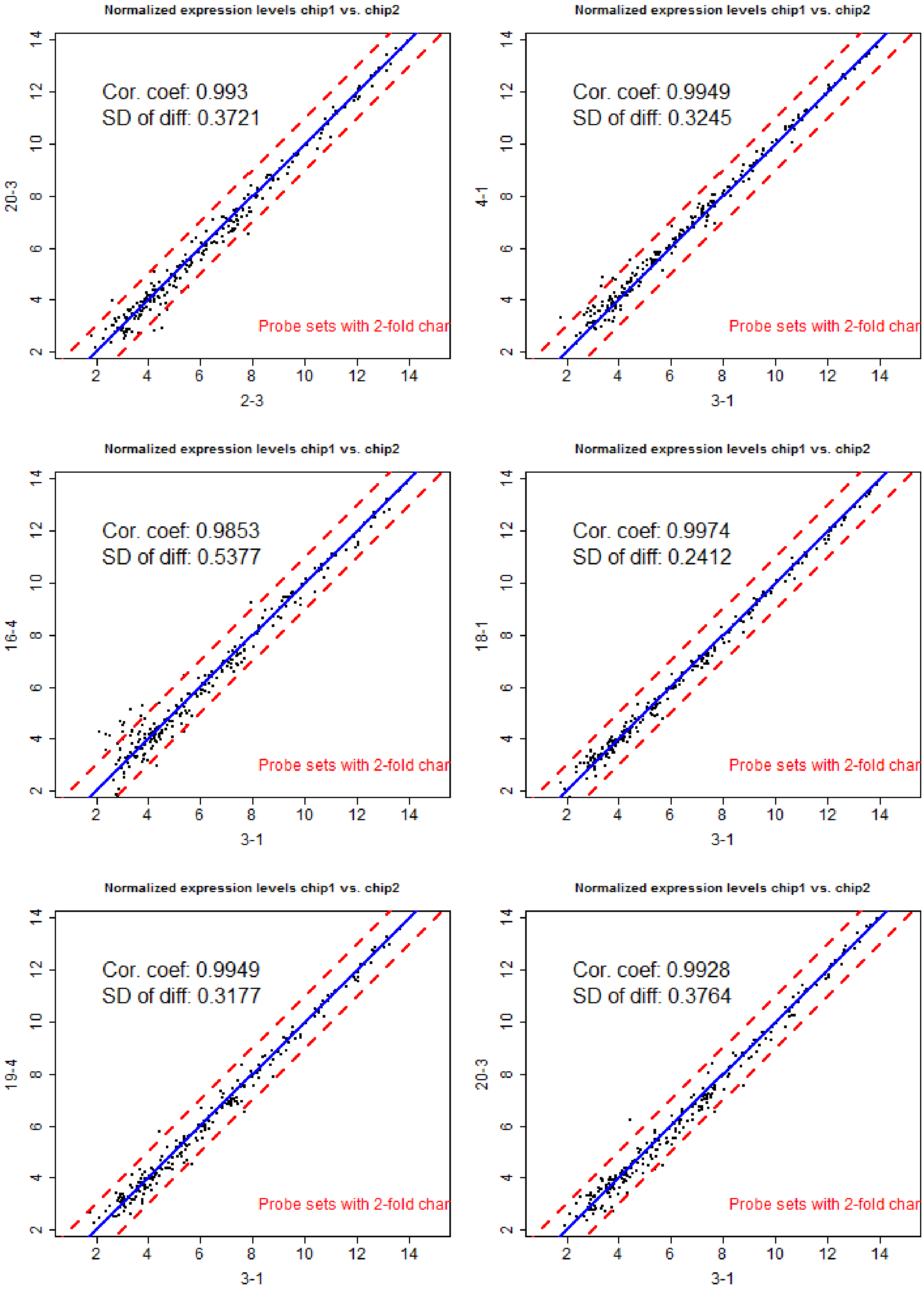
Normalized expression levels chip1 vs. chip2
Cor. coef: 0.993
SD of diff: 0.3721
Probe sets with 2-fold char
20-3
2-3
Normalized expression levels chip1 vs. chip2
Cor. coef: 0.9949
SD of diff: 0.3245
Probe sets with 2-fold char
4-1
3-1
Normalized expression levels chip1 vs. chip2
Cor. coef: 0.9853
SD of diff: 0.5377
Probe sets with 2-fold char
16-4
3-1
Normalized expression levels chip1 vs. chip2
Cor. coef: 0.9974
SD of diff: 0.2412
Probe sets with 2-fold char
18-1
3-1
Normalized expression levels chip1 vs. chip2
Cor. coef: 0.9949
SD of diff: 0.3177
Probe sets with 2-fold char
19-4
3-1
Normalized expression levels chip1 vs. chip2
Cor. coef: 0.9928
SD of diff: 0.3764
Probe sets with 2-fold char
20-3
3-1

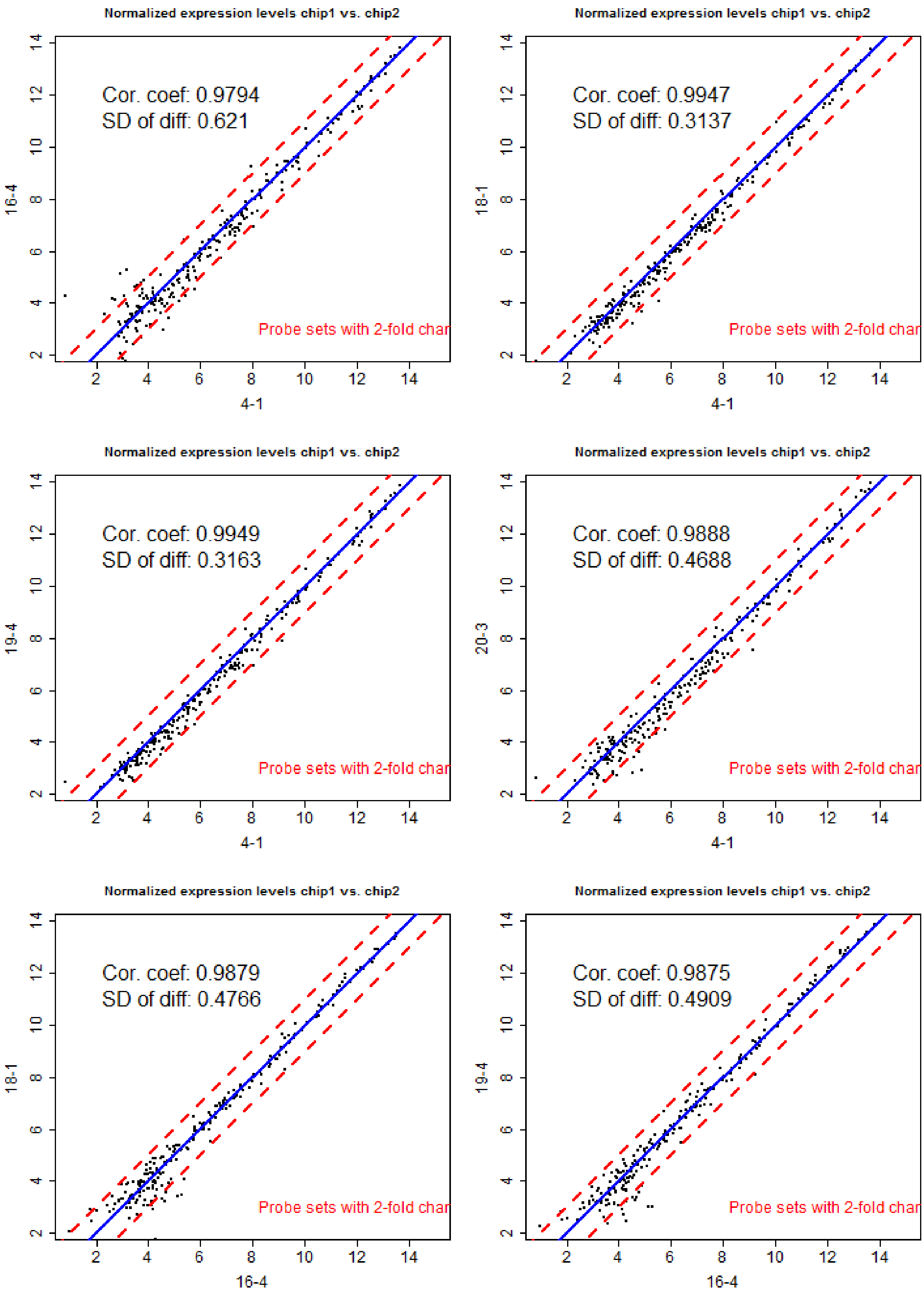
Normalized expression levels chip1 vs. chip2
Cor. coef: 0.9794
SD of diff: 0.621
Probe sets with 2-fold char
16-4
4-1
Normalized expression levels chip1 vs. chip2
Cor. coef: 0.9947
SD of diff: 0.3137
Probe sets with 2-fold char
18-1
4-1
Normalized expression levels chip1 vs. chip2
Cor. coef: 0.9949
SD of diff: 0.3163
Probe sets with 2-fold char
19-4
4-1
Normalized expression levels chip1 vs. chip2
Cor. coef: 0.9888
SD of diff: 0.4688
Probe sets with 2-fold char
20-3
4-1
Normalized expression levels chip1 vs. chip2
Cor. coef: 0.9879
SD of diff: 0.4766
Probe sets with 2-fold char
18-1
16-4
Normalized expression levels chip1 vs. chip2
Cor. coef: 0.9875
SD of diff: 0.4909
Probe sets with 2-fold char
19-4
16-4

$

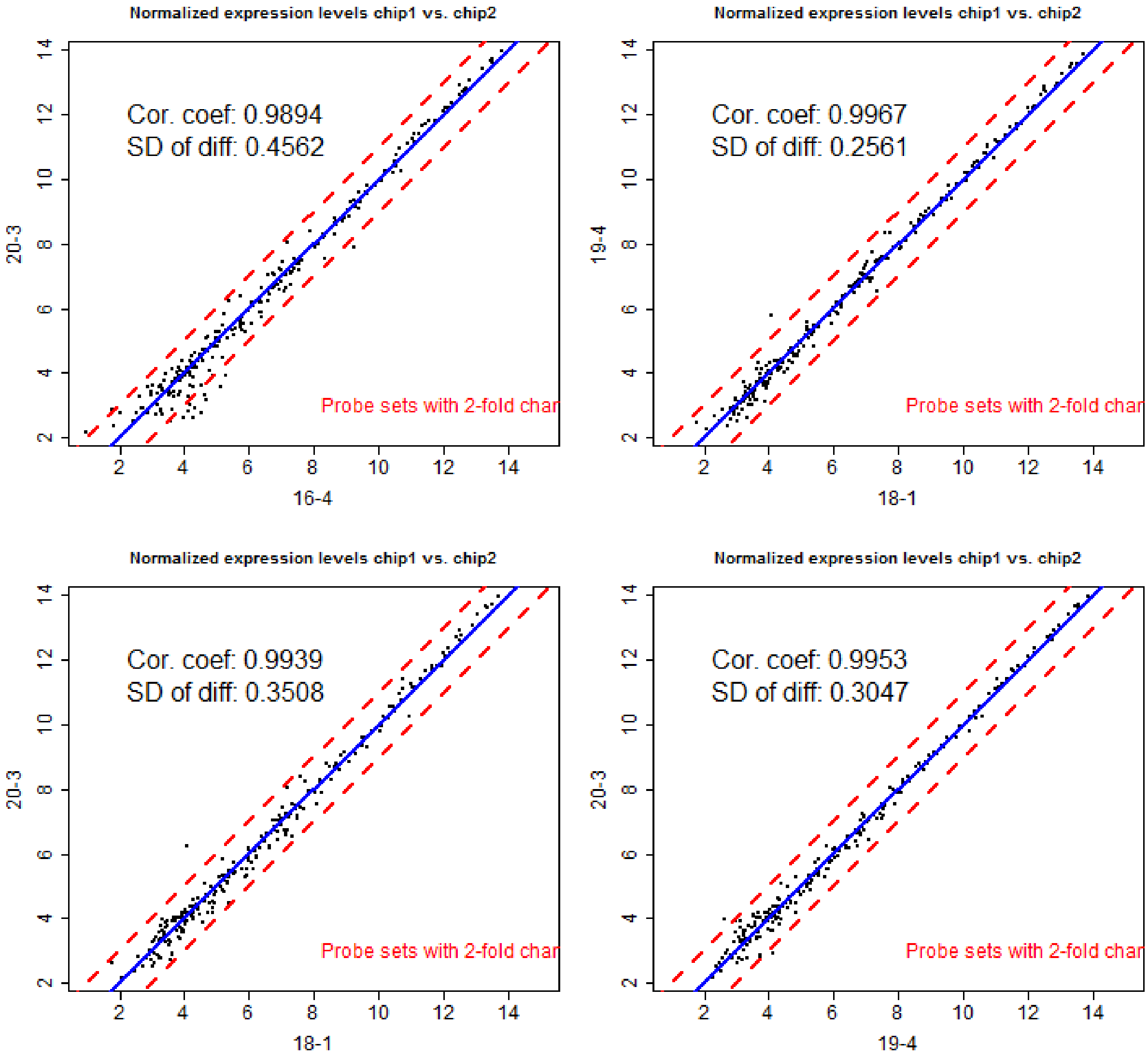
Normalized expression levels chip1 vs. chip2
Cor. coef: 0.9894
SD of diff: 0.4562
Probe sets with 2-fold char
20-3
16-4
Normalized expression levels chip1 vs. chip2
Cor. coef: 0.9967
SD of diff: 0.2561
Probe sets with 2-fold char
19-4
18-1
Normalized expression levels chip1 vs. chip2
Cor. coef: 0.9939
SD of diff: 0.3508
Probe sets with 2-fold char
20-3
18-1
Normalized expression levels chip1 vs. chip2
Cor. coef: 0.9953
SD of diff: 0.3047
Probe sets with 2-fold char
20-3
19-4

SUPPLEMENTAL TABLE 1

Gene	Forward primer	Reverse primer
Dgcr8	TCAAGGTCCGCCCTGTTTAT	GAGGCACCAAAAGGCTCACTT
Tgfβr1	ATTGCTGGTCCAGTCTGCTT	TTTTAAGGTGGTGCCCTCTG
Tgfβr2	AGTCCTTCAAGCAGACGGAT	TCACTTCTCCCACAGCGTT
Tgfβr3	CGGCTTTGGAAAAGAGAGTG	CAGGAGGAATGGTGTGGACT
Smad2	AGCAAGTGCTTGGTATGG	AGAGCAAGTGCTTGGTATGG
Smad3	CCCCCACTGGATGACTACAG	TCCATCTTCACTCAGGTAGCC
Smad4	CGCCTGTCTGAGCATTGTAC	ATTACTTGGTGGATGTTGG
Smad7	GGGTTTACAACCGCAGCAGT	GCCTTGATGGAGAAACCAGG
Mapk14	ACATCGTGTGGCAGTGAAGAAG	CTTTTGGCGTGAATGATGGA
K-Ras	GAGGCCTGCTGAAAATGACTG	ATTACTACTTGCTTCCTGTAGG
mTor	GGTGGACGAGCTCTTTGTCA	AGGAGCCCTAACACTCGGAT
Hdac8	CCAGCCACAGAAGGGATA	TTCCGTCGCAATCGTAAT
Pkrce	AAGGTGTTAGGCAAAGGCAG	GCAGCAATAGAGTTGGGTTAG
Ccnd2	TTACCTGGACCGTTTCTTGG	TGCTCAATGAAGTCGTGAGG
Rab1a	GCACTGGTTTCCAAAAATGG	GCCATGGCATCATAGTTGTG
Akt1	GTGGCAAGATGTGTATGAG	CTGGCTGAGTAGGAGAAC
FasL	ACCAACCACAGCCTTAGAGTATCATC	TGTTAAGTGGGCCACACTCCTT
Ctgf	AAGACCTGTGGGATGGGC	TGGTGCAGCCAGAAAGCTC
Sparc	CCACTCGCTTCTTTGAGACC	TAGTGGAAGTGGGTGGGGAC
Col3a1	CACCCCTCTCTTATTTTGGCAC	AGACTCATAGGACTGACCAAGGTAGTT
Fbn1	CGAGGTGACAGAGACCACAA	CTGGAGTCAAGCCAGACACA
Gapdh	AAAGCTGTGGCGTGATGG	TTCAGCTCTGGGATGACCTT
β-actin	TGTTACCAACTGGGACGACA	GGAGAGCATAGCCCTCGTAG

SUPLEMENTAL TABLE 2

	HF	ARR	CAD	CHD	VAL	FIB	HYP	MI	DIA	MC
Let-7	1		2	3	4,5	6				
Mir-15								7		
Mir-21	8, 1	9	10		11, 5	12	12	10	13	14,15
Mir-27	1	16		17, 18			19, 20		21	
Mir-29	22,1			23	24	25	26		21	
Mir-33	27					27				
Mir-98					28		19			29
Mir-99		30		31	24					
Mir-101						32	33			
Mir-193	34				24					
miR-218	35						36			
Mir-219	37				37					
miR-338			38,39							
Mir-450									40	
Mir-499		41	42	17				43,44	21	45

1 Marques, F. Z., Vizi, D., Khammy, O., Mariani, J. A. & Kaye, D. M. The transcardiac gradient of cardio-microRNAs in the failing heart. *European journal of heart failure* **18**, 1000-1008, doi:10.1002/ejhf.517 (2016).

2 Faccini, J. *et al.* Circulating miR-155, miR-145 and let-7c as diagnostic biomarkers of the coronary artery disease. *Scientific reports* **7**, 42916, doi:10.1038/srep42916 (2017).

3 Cao, L. *et al.* microRNA expression profiling of the developing mouse heart. *International journal of molecular medicine* **30**, 1095-1104, doi:10.3892/ijmm.2012.1092 (2012).

4 Jung, S. & Bohan, A. Genome-wide sequencing and quantification of circulating microRNAs for dogs with congestive heart failure secondary to myxomatous mitral valve degeneration. *Am J Vet Res* **79**, 163-169, doi:10.2460/ajvr.79.2.163 (2018).

5 Wang, H. *et al.* MicroRNA Expression Signature in Human Calcific Aortic Valve Disease. *Biomed Res Int* **2017**, 4820275, doi:10.1155/2017/4820275 (2017).

6 Wang, X. *et al.* MicroRNA Let-7i negatively regulates cardiac inflammation and fibrosis. *Hypertension* **66**, 776-785, doi:10.1161/HYPERTENSIONAHA.115.05548 (2015).

7 Hullinger, T. G. *et al.* Inhibition of miR-15 protects against cardiac ischemic injury. *Circ Res* **110**, 71-81, doi:10.1161/CIRCRESAHA.111.244442 (2012).

8 Sygitowicz, G. *et al.* Circulating microribonucleic acids miR-1, miR-21 and miR-208a in patients with symptomatic heart failure: Preliminary results. *Archives of cardiovascular diseases* **108**, 634-642, doi:10.1016/j.acvd.2015.07.003 (2015).

9 Barana, A. *et al.* Chronic atrial fibrillation increases microRNA-21 in human atrial myocytes decreasing L-type calcium current. *Circ Arrhythm Electrophysiol* **7**, 861-868, doi:10.1161/CIRCEP.114.001709 (2014).

10 Wang, F. *et al.* Atherosclerosis-related circulating miRNAs as novel and sensitive predictors for acute myocardial infarction. *PLoS One* **9**, e105734, doi:10.1371/journal.pone.0105734 (2014).

11 Kolpa, H. J. *et al.* miR-21 represses Pdcd4 during cardiac valvulogenesis. *Development* **140**, 2172-2180, doi:10.1242/dev.084475 (2013).

12 Thum, T. *et al.* MicroRNA-21 contributes to myocardial disease by stimulating MAP kinase signalling in fibroblasts. *Nature* **456**, 980-984, doi:10.1038/nature07511 (2008).

13 Shen, E., Diao, X., Wang, X., Chen, R. & Hu, B. MicroRNAs involved in the mitogen-activated protein kinase cascades pathway during glucose-induced cardiomyocyte hypertrophy. *Am J Pathol* **179**, 639-650, doi:10.1016/j.ajpath.2011.04.034 (2011).

14 Xu, H. F. *et al.* MicroRNA21 regulation of the progression of viral myocarditis to dilated cardiomyopathy. *Mol Med Rep* **10**, 161-168, doi:10.3892/mmr.2014.2205 (2014).

15 Corsten, M. F. *et al.* MicroRNA profiling identifies microRNA-155 as an adverse mediator of cardiac injury and dysfunction during acute viral myocarditis. *Circ Res* **111**, 415-425, doi:10.1161/CIRCRESAHA.112.267443 (2012).

16 Takahashi, K. *et al.* High-fat diet increases vulnerability to atrial arrhythmia by conduction disturbance via miR-27b. *J Mol Cell Cardiol* **90**, 38-46, doi:10.1016/j.yjmcc.2015.11.034 (2016).

17 Yu, K. *et al.* Association of miR-196a2, miR-27a, and miR-499 polymorphisms with isolated congenital heart disease in a Chinese population. *Genet Mol Res* **15**, doi:10.4238/gmr15048929 (2016).

18 O'Brien, J. E., Jr. *et al.* Noncoding RNA expression in myocardium from infants with tetralogy of Fallot. *Circ Cardiovasc Genet* **5**, 279-286, doi:10.1161/CIRCGENETICS.111.961474 (2012).

19 Wang, J. *et al.* Cardiomyocyte overexpression of miR-27b induces cardiac hypertrophy and dysfunction in mice. *Cell Res* **22**, 516-527, doi:10.1038/cr.2011.132 (2012).

20 Wang, Y., Chen, S., Gao, Y. & Zhang, S. Serum MicroRNA-27b as a Screening Biomarker for Left Ventricular Hypertrophy. *Tex Heart Inst J* **44**, 385-389, doi:10.14503/THIJ-16-5955 (2017).

21 Costantino, S., Paneni, F., Luscher, T. F. & Cosentino, F. MicroRNA profiling unveils hyperglycaemic memory in the diabetic heart. *Eur Heart J* **37**, 572-576, doi:10.1093/eurheartj/ehv599 (2016).

22 Naga Prasad, S. V. *et al.* A unique microRNA profile in end-stage heart failure indicates alterations in specific cardiovascular signaling networks. *PloS one* **12**, e0170456, doi:10.1371/journal.pone.0170456 (2017).

23 Zhu, S. *et al.* Identification of maternal serum microRNAs as novel non-invasive biomarkers for prenatal detection of fetal congenital heart defects. *Clin Chim Acta* **424**, 66-72, doi:10.1016/j.cca.2013.05.010 (2013).

24 Shi, J., Liu, H., Wang, H. & Kong, X. MicroRNA Expression Signature in Degenerative Aortic Stenosis. *Biomed Res Int* **2016**, 4682172, doi:10.1155/2016/4682172 (2016).

25 Van Rooij, E. *et al.* Dysregulation of microRNAs after myocardial infarction reveals a role of miR-29 in cardiac fibrosis. *Proc Natl Acad Sci U S A* **105**, 13027-13032, doi:10.1073/pnas.0805038105 (2008).

26 Sassi, Y. *et al.* Cardiac myocyte miR-29 promotes pathological remodeling of the heart by activating Wnt signaling. *Nat Commun* **8**, 1614, doi:10.1038/s41467-017-01737-4 (2017).

27 Zhang, X. & Fernandez-Hernando, C. miR-33 Regulation of Adaptive Fibrotic Response in Cardiac Remodeling. *Circ Res* **120**, 753-755, doi:10.1161/CIRCRESAHA.117.310575 (2017).

28 Li, Q., Freeman, L. M., Rush, J. E. & Laflamme, D. P. Expression Profiling of Circulating MicroRNAs in Canine Myxomatous Mitral Valve Disease. *Int J Mol Sci* **16**, 14098-14108, doi:10.3390/ijms160614098 (2015).

29 Yang, Y., Ago, T., Zhai, P., Abdellatif, M. & Sadoshima, J. Thioredoxin 1 negatively regulates angiotensin II-induced cardiac hypertrophy through upregulation of miR-98/let-7. *Circ Res* **108**, 305-313, doi:10.1161/CIRCRESAHA.110.228437 (2011).

30 Natsume, Y. *et al.* Combined Analysis of Human and Experimental Murine Samples Identified Novel Circulating MicroRNAs as Biomarkers for Atrial Fibrillation. *Circ J*, doi:10.1253/circj.CJ-17-1194 (2018).

31 Kehler, L., Biro, O., Lazar, L., Rigo, J., Jr. & Nagy, B. Elevated hsa-miR-99a levels in maternal plasma may indicate congenital heart defects. *Biomed Rep* **3**, 869-873, doi:10.3892/br.2015.510 (2015).

32 Pan, Z. *et al.* MicroRNA-101 inhibited postinfarct cardiac fibrosis and improved left ventricular compliance via the FBJ osteosarcoma oncogene/transforming growth factor-beta1 pathway. *Circulation* **126**, 840-850, doi:10.1161/CIRCULATIONAHA.112.094524 (2012).

33 Wei, L. *et al.* MicroRNA-101 inhibits rat cardiac hypertrophy by targeting Rab1a. *J Cardiovasc Pharmacol* **65**, 357-363, doi:10.1097/FJC.0000000000000203 (2015).

34 Wong, L. L. *et al.* Circulating microRNAs in heart failure with reduced and preserved left ventricular ejection fraction. *Eur J Heart Fail* **17**, 393-404, doi:10.1002/ejhf.223 (2015).

35 Chen, Y. *et al.* Detailed characterization of microRNA changes in a canine heart failure model: Relationship to arrhythmogenic structural remodeling. *J Mol Cell Cardiol* **77**, 113-124, doi:10.1016/j.yjmcc.2014.10.001 (2014).

36 Liu, J. J. *et al.* miR-218 Involvement in Cardiomyocyte Hypertrophy Is Likely through Targeting REST. *Int J Mol Sci* **17**, doi:10.3390/ijms17060848 (2016).

37 Yang, V. K. *et al.* Circulating exosome microRNA associated with heart failure secondary to myxomatous mitral valve disease in a naturally occurring canine model. *J Extracell Vesicles* **6**, 1350088, doi:10.1080/20013078.2017.1350088 (2017).

38 Zhang, H. *et al.* Profiling of differentially expressed microRNAs in arrhythmogenic right ventricular cardiomyopathy. *Sci Rep* **6**, 28101, doi:10.1038/srep28101 (2016).

39 Xiao, J. *et al.* MicroRNA expression signature in atrial fibrillation with mitral stenosis. *Physiol Genomics* **43**, 655-664, doi:10.1152/physiolgenomics.00139.2010 (2011).

40 Chavali, V., Tyagi, S. C. & Mishra, P. K. Differential expression of dicer, miRNAs, and inflammatory markers in diabetic Ins2+/- Akita hearts. *Cell Biochem Biophys* **68**, 25-35, doi:10.1007/s12013-013-9679-4 (2014).

41 Ling, T. Y. *et al.* Regulation of the SK3 channel by microRNA-499--potential role in atrial fibrillation. *Heart Rhythm* **10**, 1001-1009, doi:10.1016/j.hrthm.2013.03.005 (2013).

42 Oerlemans, M. I. *et al.* Early assessment of acute coronary syndromes in the emergency department: the potential diagnostic value of circulating microRNAs. *EMBO Mol Med* **4**, 1176-1185, doi:10.1002/emmm.201201749 (2012).

43 Adachi, T. *et al.* Plasma microRNA 499 as a biomarker of acute myocardial infarction. *Clin Chem* **56**, 1183-1185, doi:10.1373/clinchem.2010.144121 (2010).

44 D'Alessandra, Y. *et al.* Circulating microRNAs are new and sensitive biomarkers of myocardial infarction. *Eur Heart J* **31**, 2765-2773, doi:10.1093/eurheartj/ehq167 (2010).

45 Corsten, M. F. *et al.* Circulating MicroRNA-208b and MicroRNA-499 reflect myocardial damage in cardiovascular disease. *Circ Cardiovasc Genet* **3**, 499-506, doi:10.1161/CIRCGENETICS.110.957415 (2010).
